# Estimation of the within-herd transmission rates of bovine viral diarrhoea virus in extensively grazed beef cattle herds

**DOI:** 10.1186/s13567-019-0723-2

**Published:** 2019-11-29

**Authors:** Jun-Hee Han, Jenny F. Weston, Cord Heuer, M. Carolyn Gates

**Affiliations:** 1 EpiCentre, School of Veterinary Science, Massey University, Private Bag 11-222, Palmerston North, New Zealand; 2 School of Veterinary Science, Massey University, Private Bag 11-222, Palmerston North, New Zealand

## Abstract

Many research groups have developed mathematical models to simulate the dynamics of BVDV infections in cattle herds. However, most models use estimates for within-herd BVDV transmission rates that are either based on expert opinion or adapted from other dairy herd simulation models presented in the literature. There is currently little information on the transmission rates for BVDV in extensively grazed beef herds partly due to the logistical challenges in obtaining longitudinal data of individual animal’s seroconversion, and it may not be appropriate to apply the same transmission rates from intensive dairy herds given the significant differences in herd demographics and management. To address this knowledge gap, we measured BVDV antibody levels in 15 replacement heifers in each of 75 New Zealand beef breeding farms after their first calving and again at pregnancy scanning or weaning to check for seroconversion. Among these, data from 9 farms were used to infer the within-herd BVDV transmission rate with an approximate Bayesian computation method. The most probable within-herd BVDV transmission rate was estimated as 0.11 per persistently infected (PI) animal per day with a 95% highest posterior density interval between 0.03 and 0.34. This suggests that BVDV transmission in extensively grazed beef herds is generally slower than in dairy herds where the transmission rate has been estimated at 0.50 per PI animal per day and therefore may not be sufficient to ensure that all susceptible breeding females gain adequate immunity to the virus before the risk period of early pregnancy for generating new PI calves.

## Introduction

Bovine viral diarrhoea virus (BVDV) is recognised for its significant impacts on cattle health, welfare and production worldwide [1]. An important epidemiological feature of BVDV is that if susceptible dams are infected in early-mid gestation before the fetus has developed a competent immune system, the fetus will become persistently infected (PI) with BVDV and the resulting calf will shed large quantities of the virus throughout its life-time [2]. Since PI animals act as the primary reservoir for BVDV transmission in cattle populations, most BVDV control programmes are therefore focused on identifying and eliminating existing PI animals in a timely fashion as well as preventing the creation of new PI animals [3]. This can be accomplished through various interventions such as conducting animal- or herd-level diagnostic testing, vaccinating susceptible animals, and improving farm biosecurity [4].

To appraise the economic argument for implementing BVDV control measures, several research groups have developed mathematical simulation models to explore BVDV transmission dynamics and its impact on production at varying scales (e.g. in a farm or multiple farms in a region/country) [5–7]. In these models, the infection of a susceptible individual is determined by the force of infection, which is a function of the numbers of PI and transiently infected (TI) individuals at a given time and the transmission rates ($\beta$) for both types of infected cattle. Given the definition of transmission rate is *per capita* rate at which two individuals have an effective contact (physically close contact with sufficient time that disease transmission could occur if there was an infectious individual) [8], PI animals are typically assigned larger $\beta$ value than TI animals due to their higher viral shedding rates. Consequently the predictions of simulation models are often highly sensitive to the $\beta$ value for PI animals [9].

An interesting point about BVDV modelling studies with respect to the transmission rates for PI animals is that most studies used parameter values based on either expert opinion or the values assumed by other simulation models [10]. One of the most commonly used within-herd BVDV transmission rates for PI animals was suggested by Viet et al. [7], who set the value to 0.5 per PI animal per day (using frequency-dependent assumption) based on other reports [11, 12]. Although it was not estimated from empirical data, the suggested value successfully explained the BVDV spread on a dairy farm, and their method has been reproduced and adopted by subsequent modellers who simulated BVDV spread under their own epidemiological circumstances [9, 13–16]. However, as briefly pointed out in the original paper, a robust transmission rate should be estimated based on longitudinal observations of individual BVDV infection status while considering other management factors, such as production type, population size or density, or herd structure, since those factors can affect BVDV transmission rates within individual herds [14, 17]. Therefore, applying the same $\beta$ value for simulating BVDV transmission in other populations with substantially different management features such as extensively grazed beef cattle may not be appropriate.

To date, most of the reports on BVDV seroconversion have been based on the dairy industry, whereas only a limited number of studies have focused on the extensively grazed beef industry due in part to the logistical challenges that make it difficult to collect serial samples of individual animals to measure their seroconversion status [18]. The transmission of BVDV in extensively grazed beef herds is expected to be different than in intensive dairy herds since (1) the beef cattle are commonly grazed over a vast area and not often gathered during a year so that the chance of having effective contacts is likely to be lower, while (2) new born calves stay with their dams until weaning (6–7 months), hence susceptible dams could receive continued exposure to new born PI calves during the breeding period, which likely increases the chance of generating new PI calves in the following calving season.

As part of a larger research programme to address the knowledge gaps around BVDV transmission in extensively grazed New Zealand beef herds, we conducted a longitudinal study to measure BVDV antibody levels in 15 first-calf heifers (replacement heifers immediately after their first calving and before being mixed to the adult cow herd) before and after subsequent breeding period to check for the evidence of seroconversion. Although BVDV transmission rates can be roughly estimated by fitting the change in number of BVDV antibody positive animals over time with a generalised linear regression model [19], this method requires detection of all seroconversions based on a series of observations on every animal’s BVDV infection status in a herd [11]. Given the limited number of observations in our study, we estimated the transmission rate using an approximate Bayesian computation (ABC) methods. Briefly, ABC is a set of Bayesian methods that infer the posterior distribution of parameters by randomly drawing a sample of the parameters from an initial distribution to simulate data and accepting only the sample of which the simulated data is close enough to the observed data according to a pre-defined distance (or tolerance) [20].

Using the seroconversion data from our longitudinal field studies, the objective of this current study was to infer the within-herd BVDV transmission rate for PI animals ($\beta_{P}$) in beef breeding farms using an ABC method while accounting for differences in the herd management structures.

## Materials and methods

### Data collection and extraction

A longitudinal observational field study was conducted from September 2017 to September 2018 to estimate the proportion of first-calf heifers that were still susceptible to BVDV prior to their second breeding and to estimate the rates of seroconversion to BVDV during the breeding/early pregnancy period. A total of 75 commercial beef breeding farms from different regions across New Zealand were recruited by convenience sampling through 10 participating veterinary clinics. The selection criteria were that the farms (1) were not currently vaccinating replacement breeding females against BVDV since vaccination is known to interfere with antibody-based screening tests for BVDV, (2) had at least 15 replacement heifers due to calve in the 2017/2018 calving season (generally between August 2017 and November 2017), and (3) were willing to yard animals at two time points for sampling (once prior to the second breeding (i.e. first sampling event) and again at pregnancy scanning or weaning (i.e. second sampling event)).

For each enrolled farm, a participating veterinarian collected blood samples from 15 randomly selected first-calf heifers with calves at foot before the breeding period and recorded the ear tag IDs for each individual animal. The samples were then transported to a commercial veterinary diagnostic laboratory and analysed individually using a BVDV antibody ELISA test (IDEXX BVDV Total Ab Test; IDEXX Laboratories Inc., Westbrook MA, USA). The samples were considered as BVDV antibody test-positive if the sample to positive (S/P) ratio was > 0.17 which was determined by the laboratory (cut-off value by the manufacturer was 0.2). A survey was administered to farmers at the time of sampling to collect information about general management practices including the date of management events (breeding, weaning, and calving) and demographic features (herd structure, herd size, and age of heifers when they first calve). A copy of the complete survey is provided in Additional file 1.

Based on the test results from the first sampling event, BVDV antibody negative heifers were identified, and the participating veterinarians were asked to re-collect blood sample from each negative animal at either pregnancy scanning or weaning. The samples were transported to the same diagnostic laboratory for analysis using the same ELISA test, and the same cut-off value (S/P > 0.17) was used to identify whether animals had become BVDV antibody positive. We assumed that a change in status from negative to positive indicated seroconversion to BVDV, so only the results of farms with increased number of BVDV test-positive heifers in the second sampling event were extracted for further study.

### Simulation model

In order to estimate the $\beta_{P}$ values using an ABC algorithm, we first developed a stochastic, individual-based BVDV transmission simulation model to replicate BVDV dynamics in a typical extensively-grazed New Zealand beef herd. For most New Zealand beef farms, weaning occurs at approximately 6 to 7 months of age [21]. Initial breeding occurs at approximately 14 to 15 months of age with first calving at approximately 24 months of age on most of beef farms, while some farmers breed heifers for the first time at approximately 26 to 27 months old with them calving at approximately 36 months old. The breeding period usually lasts from 8 to 12 weeks. Based on the survey result, most farmers kept the replacement heifer herd separate from the adult breeding cows even after their first calving.

Since the exact mixing date of replacement breeding heifers with adult breeding cows for each eligible farm was unknown, we assumed that the mixing coincided with pregnancy scanning of replacement breeding heifers (and weaning of their first calves) so that the replacement heifer herd had been grazed separately (except from their calves) for the whole study period since their weaning. Accordingly, the model only included two demographic groups; the replacement heifers and their calves at foot. We assumed that no other contacts with other groups of animals on the farm occurred during this time period, and BVDV transmission from breeding bulls was not considered given that the farmers reported using BVDV vaccinated or BVDV-free certified breeding bulls.

The simulation model started at the day of weaning for the replacement heifers (day 0) and followed individual animals through their first year of life, initial breeding period, their first calving, the day of first BVDV blood sampling event, second breeding period, and the day of second sampling event (pregnancy scanning or weaning). The total number of heifers and the calendar dates for any management events were taken directly or calibrated from the survey data provided by the farmers to make the models as herd-specific as possible. The calves-at-foot group was only modelled from birth until weaning.

BVDV status of animals in the simulation model was either; immune via maternal antibody (M; only for the calves-at-foot), susceptible (S), transiently infected (T), persistently infected (P), and recovered (R). BVDV transmission was assumed to be frequency-dependent under homogeneous mixing. Since we did not assess the BVDV status of the entire herd nor conduct any antigen testing to confirm the presence of PI animals, we could not actually determine when the tested heifers were exposed to BVDV or the source of their exposure. Given that BVDV is endemic in New Zealand [18], we therefore assumed that a random proportion of replacement heifers in each farm ($\mu_{i}$) were recovered and seropositive to BVDV at the time of weaning (day 0) due to the anamnestic response (if calves with enough maternal antibody were exposed to BVDV, they show a higher immune response to BVDV when they are re-exposed to the virus after the depletion of maternal antibody) [22–25]. We then simulated from the day of weaning (day 0) and introduced BVDV via PI animals at a random point of time between day 0 and the day of first sampling event ($\tau_{i}$) for each farm. The introduction of PI animals in the simulation model was conducted by converting a random proportion of heifers as PI ($\rho_{i}$). The introduction of BVDV via TI animals was not considered as they have been demonstrated to have a limited role in BVDV transmission [26]. Also, it was assumed that the introduction of PI animals occurred at a single time only, and that 50% of the introduced PI animals had been removed annually from the point of introduction [6].

The probability of BVDV infection for each susceptible animal (*Prob*) at a given day was;

$$Prob = 1 - e^{{ - \lambda_{t} }}$$

$$\lambda_{t} = \beta_{P} \frac{{P_{t} }}{N} + \beta_{T} \frac{{T_{t} }}{N}$$

where $\lambda_{t}$ is the force of BVDV infection at day *t*, $P_{t}$ and $T_{t}$ are the number of PI and TI animals, respectively, at day *t*, *N* is the herd size, and $\beta_{T}$ is the within-herd BVDV transmission rate for TI animals. Since our aim was to estimate $\beta_{P}$, $\beta_{T}$ was assumed to be proportionally (0.05) lower than $\beta_{P}$ with the proportion being calibrated based on other studies [10, 27]. One should note that the meaning of transmission rates above is actually the number of effective contacts per infectious individual per day (i.e. effective contact rate), which is different from the classic definition of transmission rate [8]. However, we used the effective contact rate and transmission rate interchangeably for the coherence with other BVDV modelling works. TI animals were assumed to recover and became seropositive after *x* days of infection, where *x* was randomly chosen between 10 and 20 days [28–30]. Once recovered, animals were assumed to have protective immunity against BVDV for the remainder of the simulation given the short duration of simulation.

Each simulation started by establishing a female herd of $N_{i}$ weaned animals, where *N* was the reported herd size of replacement breeding heifers via survey for farm *i*. Puberty in heifers was assumed to start at 380 days of age [31] with oestrus cycles occurring every 18 to 24 days thereafter until the animal became pregnant. When a pregnant heifer calved or had an abortion, the next oestrus was assumed to occur after 15 to 49 days [32] and assumed to be a silent oestrus. When the simulation reached to the start date of initial breeding period which was calibrated based on the date of breeding and age of heifers to calve in the survey, heifers were mated with bulls and only the heifers in oestrus were conceived with the probability of 62.0% during the breeding period [33]. The gestation period was 281 days and the natural abortion rate was set at 0.0001 per day over the whole gestation period to achieve a total abortion rate of 3.5% for the season [34, 35]. For each new born calves from heifers, 0.0009 per day of natural mortality rate was applied until weaning (86.7% of calves surviving to 180 days) [36].

The probability of abortion of replacement heifers due to BVDV infection was varied by the days of pregnancy at the time of infection (Table 1). Likewise, the BVDV infection status of new born calves was determined by the stage of pregnancy the heifers were in when they became infected with BVDV. The period of maternal BVDV antibody protection for individual calves born to recovered replacement heifers was randomly selected from a normal distribution with the mean and standard deviation of 155 and 31 days, respectively [37, 38] (Additional file 2).

**Table 1:** **Definition and value of parameters used for within-herd BVDV transmission model**

Parameter definitions	Value	References
Oestrus cycle (day)	*U*(18, 24)	[31]
Probability of conception	0.62	[33]
Natural abortion rate	0.0001 per day	[34, 35]
Natural calf mortality rate until weaning	0.0009 per day	[36]
$\beta_{P}$: Within-herd BVDV transmission rate (per PI animal per day)	To be estimated	
$\beta_{T}$: Within-herd BVDV transmission rate (per TI animal per day)	$\beta_{P} \times 0.05$	[10, 27]
Infectious period of TI animals (day)	*U*(10, 20)	[28–30]
Period of maternal BVDV antibody protection (day)	*N*(155, 31^2^)	[37, 38]
Mortality rate of PI animals	0.0019 per day	[6, 9]
Probability of abortion if infected during early pregnancy (day 0–41)	0.8	[52, 53]
Probability of abortion if infected during mid-pregnancy (day 42–150)	0.25	[52, 53]
Probability of calving PI animal if infected during mid-pregnancy	0.934	[2, 52]
Probability of calving an immuned calf if infected during mid-pregnancy	0.033	[2, 52]

Key: *N*(·,·), normal distribution(mean, variance); *U*(·,·), uniform distribution(lower limit, upper limit).

**Figure 1:**
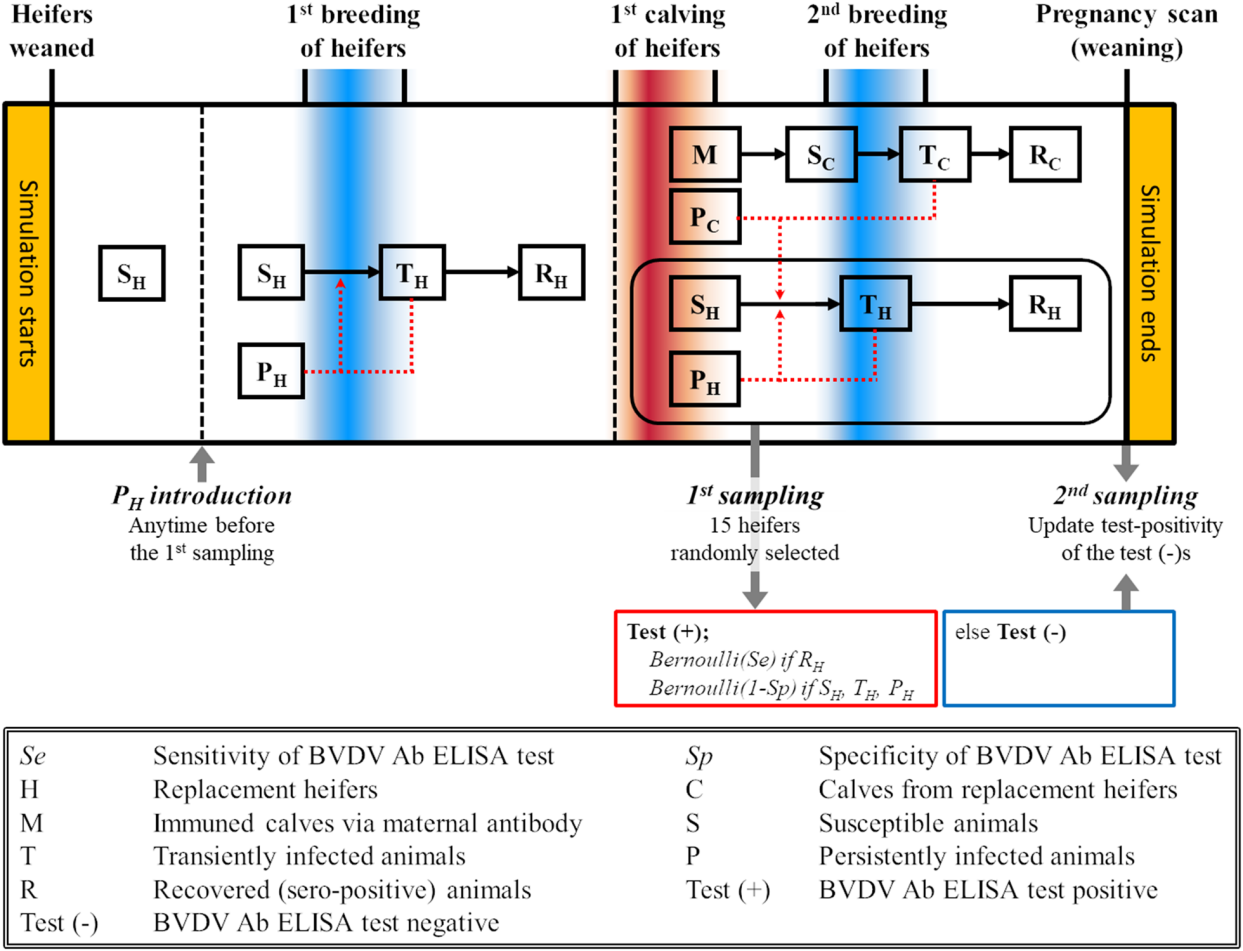
**Description of within-herd simulation for estimating within-herd BVDV transmission rate.** Blue and red shaded areas indicate the breeding and calving periods, respectively. Red dotted arrow represents the contribution to the force of BVDV infection.

At the day of first sampling event, 15 individuals were selected randomly from the simulated heifers and BVDV ELISA antibody test-positivity of each individual was probabilistically determined based on each animal’s BVDV infection status, test sensitivity (0.99), and specificity (0.81) [39]. Each simulation ended when it reached the day of second sampling event, where similar to the field studies, any of the individual animals that tested negative for BVDV antibodies were re-sampled to determine whether they had seroconverted. An illustration of the simulation is provided in Figure 1 and the definition and values for the model parameters are shown in Table 1.

### Estimation of within-herd BVDV transmission rates

Along with $\beta_{P}$, there was no information about the initial proportion of BVDV seropositive animals ($\mu_{i}$), the proportion of introduced PI animals ($\rho_{i}$), and the day of PI animals being introduced ($\tau_{i}$) in each study herd *i*. Given the partial observation of whole BVDV transmission in this study with the intractable likelihood of the simulation model, we estimated parameters using an ABC method. Specifically, we chose an approximate Bayesian computation-sequential Monte Carlo (ABC-SMC) algorithm to achieve the computational efficiency [20, 40]. In the ABC-SMC, parameters are inferred through a series of estimation sequences (or SMC sequence), each of which has a decreased distance threshold (ε) than its previous sequence. A SMC sequence starts with selecting a parameter value based on the perturbation of estimated values in the previous sequence (or the random sampling of a prior distribution in case of the first SMC sequence), and applying the selected value to a model to generate data (i.e. simulated data). The difference between the observed and simulated data is measured by comparing the summary statistics of each data (i.e. distance), and only the value showing the distance less than a threshold of the sequence is retained. Current SMC sequence ends when a number of values (i.e. particle) are collected, and the whole process repeats until the particles of final SMC sequence are collected. The distribution drawn by the particles of final sequence is a marginal posterior distribution, which approximates the posterior distribution of the parameter given the observed data. Since it is a modeller’s choice which summary statistics to use to measure the distance, the chosen summary statistics should contain sufficient information about the parameter because it affects the posterior distribution of the parameter with only the reduced information of each data [41]. The detailed algorithm for the ABC-SMC is provided in Additional file 3.

We assumed that all study herds shared the common distribution of $\beta_{P}$ while other parameters varied by the herds. For the prior distribution of within-herd BVDV transmission rate for PI animals ($\uppi\left( {\beta_{P} } \right)$), we assumed that the value was less than 0.5 with mode of 0.25 (with 60% certainty). The mode of the prior distribution was set to achieve 60% of susceptible animals being infected by a PI animal in a year [42], however, the distribution was still wide enough to cover various values between 0 and 1. A uniform prior between 0 and 1 was used for the initial proportion of BVDV seropositive animals ($\uppi(\mu_{i} )$). The prior distribution of the proportion of introduced PI animals ($\uppi\left( {\rho_{i} } \right)$) followed a beta distribution, with a certainty of 50% that the chosen value was less than 0.1 (with the mode at 0.02). The prior of the day of PI animals being introduced ($\uppi\left( {\tau_{i} } \right)$) followed a uniform distribution, as it was randomly selected between 0 (i.e. PI animals had been co-grazed with the heifers from their weaning onward) and the number of days from day 0 to the day of first sampling event (i.e. PI animals were introduced at the day of first sampling event). All priors were assumed to be independent.

For the perturbation kernel, a component-wise Gaussian kernel with the variance as 0.68 times of the variance of particles in the previous SMC sequence was used [43]. We used two summary statistics to estimate the distance ($D_{k}$) between the observed and simulated data; (1) the observed ($Obs_{1}$) and simulated ($Sim_{1}$) number of test positive heifers in the first sampling round ($D_{1}$), and (2) the observed ($Obs_{2}$) and simulated ($Sim_{2}$) number of test positive heifers (among those were test negative at the first round) in the second sampling round ($D_{2}$). Each summary statistics was calculated as below;

$$D_{k} = \sqrt {\mathop \sum \limits_{i = 1}^{n} (Obs_{k}^{i} - Sim_{k}^{i} )^{2} }$$

where $n$ is the number of herds in this study. For each SMC sequence, the simulation model was iterated until 2000 acceptable particles were generated, and the acceptable distance thresholds were set as 50^th^ percentile of the distances of accepted particles in the previous sequence. The marginal posterior distribution of each parameter was achieved after 15 SMC sequences (Table 2).

**Table 2:** **Prior distribution of parameters estimated using ABC-SMC algorithm**

Parameter	Prior distribution
Within-herd BVDV transmission rate for PI animals (*β*_*P*_)	Beta (1.18, 1.54)
Initial proportion of BVDV seropositive animals (*μ*)^a^	Uniform (0, 1)
Proportion of introduced PI animals (*ρ*)^a^	Beta (1.14, 7.79)
Day of PI animals being introduced (*τ*)^a^	Uniform (0, k)

^a^Sampled values varied by herd.

Key: k, number of days from weaning of replacement heifers to the day of first sampling event.

### Validation of the estimated parameters

Once the posterior distributions were retrieved, we inspected the fitness of the estimated parameters by reproducing BVDV transmission within study herds with the estimated parameter values while considering management features of each herd. To do this, we randomly sampled 2000 sets of parameter values from the respective posterior distributions, and applied to the simulation model to draw a distribution of simulated summary statistics ($Sim_{1}$ and $Sim_{2}$). We then visually examined whether the observed statistics located within a 95% prediction interval of the simulated summary statistics [41, 44].

We also conducted a sensitivity analysis to investigate the impact of using different prior distribution of unknown parameters. Before the sensitivity analysis, we preliminarily examined which parameter affected the most to the summary statistics in an individual farm. It showed that the statistics were the most sensitive to the values of $\mu$ and $\rho$, while other parameters had only a limited impact (see Additional file 4). Since there was a lack of information to estimate an informative $\uppi(\mu_{i} )$ in this study, we tested two different distributions (e.g. uniform distribution between 0 and 1, and normal distribution with mean of 0.3 and standard deviation of 0.1) of only the $\uppi\left( {\rho_{i} } \right)$ for the sensitivity analysis. The disease simulations were implemented in the C programming language and ABC-SMC algorithm was run in R [45].

## Results

### Longitudinal data

Overall, blood samples from 1116 individual heifers on 75 farms were collected at the first sampling event, and, of these, all heifers from 12 farms were BVDV antibody positive and 729 heifers from 63 farms tested negative for BVDV antibodies. Farms where all sampled heifers tested positive were excluded from follow-up, and we were only able to obtain the second blood samples from 673/729 heifers (92.3%) located on 55/63 farms due to logistical issues with being able to yard the same animals for re-sampling (one to five heifers were unavailable to follow-up on 19 of the 63 farms).

From the 55 re-sampled farms, all heifers from 25 farms were BVDV antibody test-negative for both sampling events, indicating no circulation of BVDV before and during the study period. Test results from 17 farms showed no additional BVDV antibody test-positive heifers possibly due to early culling/moving of PI animals, and only the 13 remaining farms indicated BVDV seroconversion. The data from these 13 farms were retained for further use in this study, however, records from 4 of these farms were discarded either because some of the animals were later found to have been BVDV vaccinated or because the second sampling event took place outside of the time-window specified for the study. Overall, test results of 133 heifers from 9 farms were used to estimate $\beta_{P}$ values. Descriptive statistics on the number of sampled and test-positive heifers at each sampling, and other management features, such as herd size, breeding period, and day of both sampling events, for each farm are provided in Additional file 5.

### Parameters estimation

**Figure 2:**
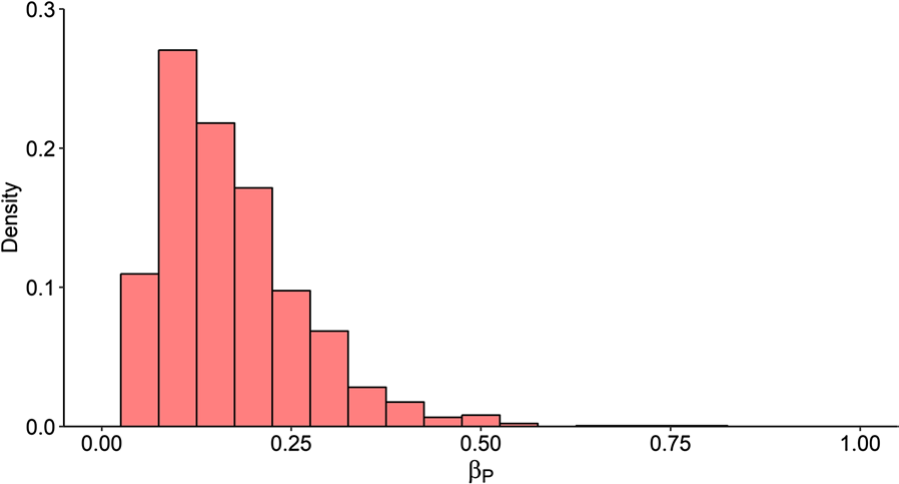
**The posterior distribution of within-herd BVDV transmission rates for PI animals (**$\varvec{\beta}_{\varvec{P}}$**).** The mode was 0.11 with a 95% highest posterior density interval between 0.03 and 0.34.

**Figure 3:**
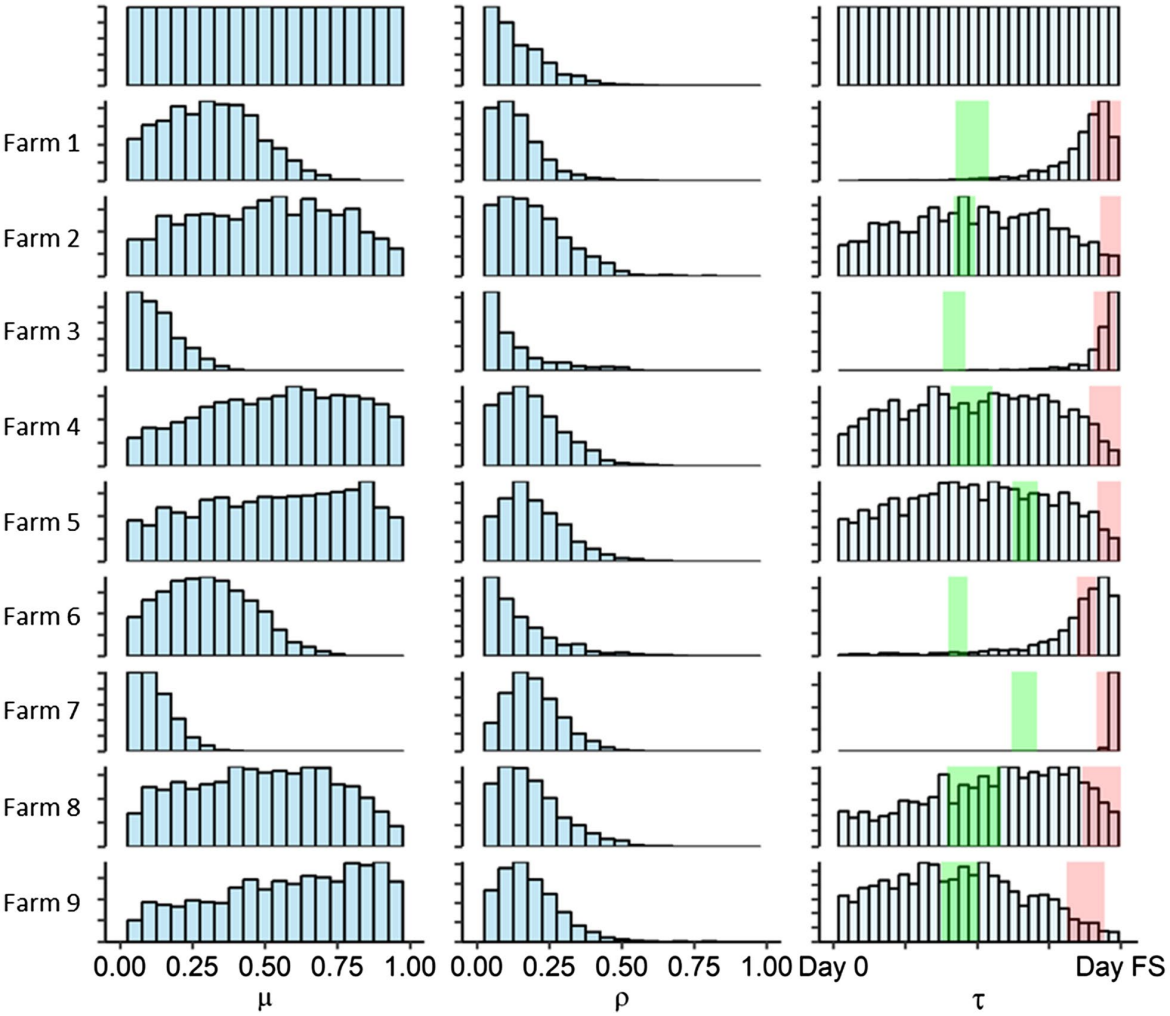
**The posterior distribution of the estimated parameters for each herd (from the second to the last row).** Left: the initial proportion of BVDV seropositive animals ($\mu$), Middle: the proportion of introduced PI animals ($\rho$), Right: the day of PI animals being introduced ($\tau$). The distributions of the first row illustrate the prior distribution of each parameter. Green and red shaded areas indicate the first breeding and calving period, respectively. Day FS indicates the day of first sampling.

The posterior distribution of $\beta_{P}$ is illustrated in Figure 2 and the evolution of $\beta_{P}$ values over 15 ABC-SMC sequences is provided in Additional file 6. The mode of posterior $\beta_{P}$ was 0.11 with a 95% highest posterior density (HPD) interval between 0.03 and 0.34 per PI animal per day. The posterior distributions of estimated $\mu , \rho , {\text{and}} \tau$ are illustrated in Table 3 and Figure 3, and the evolution of parameter values along the SMC sequences is provided in Additional file 6. The distributions of $\mu$ and $\rho$ varied between herds, with the mode of $\mu$ ranged between 0.07 and 0.84, and the mode of $\rho$ between 0.03 and 0.16. The estimated day when PI animals were first introduced varied by herd as well. The most probable (mode) day for Farms 1, 3, 6, and 7 occurred during or immediately after the first calving period, while the day for Farm 2 and 9 were during the first breeding period. The estimated day for the remaining farms were widely dispersed between the day of heifers being weaned and the first breeding period.

**Table 3:** **Mode (95% highest posterior density interval) of the estimated initial proportion of BVDV seropositive animals (μ), the proportion of introduced PI animals (ρ), and the day of PI animals being introduced (τ) for each studied farm**

Farm	$\mu$	$\rho$	$\tau$
1	0.32 (0.00, 0.58)	0.11 (0.01, 0.32)	546 (359, 590)
2	0.53 (0.05, 0.93)	0.10 (0.00, 0.42)	252 (13, 499)
3	0.07 (0.00, 0.29)	0.03 (0.00, 0.40)	525 (380, 533)
4	0.62 (0.10, 1.00)	0.15 (0.00, 0.39)	210 (24, 530)
5	0.80 (0.07, 0.98)	0.14 (0.00, 0.39)	378 (18, 866)
6	0.26 (0.00, 0.56)	0.04 (0.00, 0.41)	588 (225, 625)
7	0.08 (0.00, 0.25)	0.16 (0.03, 0.37)	945 (913, 950)
8	0.48 (0.00, 0.88)	0.12 (0.00, 0.40)	357 (53, 597)
9	0.84 (0.09, 0.98)	0.15 (0.01, 0.38)	294 (9, 543)

### Validation

The observed and simulated summary statistics for each study herd are provided in Additional file 6. For both $Sim_{1}$ and $Sim_{2}$, observed summary statistics for all 9 herds were located within a 95% prediction interval of the simulated summary statistics. Most of the observed summary statistics were located near the mode of simulated summary statistics, however, the observed $Sim_{2}$ of some but not all herds were located near the margin of 95% prediction interval (see Additional file 6). In the sensitivity analysis, some but not all parameters showed different posterior distributions when either a uniform or normal distribution of $\uppi\left( {\rho_{i} } \right)$ was used. Especially, the posterior distribution of $\rho_{i}$ was generally affected by the prior distribution of itself, indicating that the parameter $\rho_{i}$ in our model was not identifiable in general (see Additional file 7).

## Discussion

Using an ABC-SMC algorithm, we estimated the within-herd BVDV transmission rates for PI animals based on field data collected from extensively grazed beef herds. Although the ABC method has been used to estimate the transmission rate of other animal diseases, such as bovine tuberculosis [43], Peste des petits ruminants [46], and African swine fever [47], our study is the first attempt to use this method for estimating $\beta_{P}$ values to the best of our knowledge. This is most likely due to the paucity of BVDV seroconversion data, especially in extensively grazed beef herds. Other studies have used a commonly reported $\beta_{P}$ value from intensively managed dairy herds to explain BVDV spread [9, 13–16]. However, more efforts should be made to estimate robust $\beta_{P}$ values based on empirical data that take into consideration other herd-level management factors that can affect BVDV transmission.

Estimation of $\beta_{P}$ values was based on only 9 out of 75 initially recruited farms, and we acknowledge the possibility of selection bias in this study. While it is possible that the 12 farms that were not followed-up at the second sampling event had higher $\beta_{P}$ values than the estimated values in this study, it is also plausible that the heifers on those farms had been exposed to the virus for much longer time periods (e.g. co-grazed with PI calves from birth) or that there were a larger number of PI animals compared to other farms that could have led to more efficient within-herd BVDV transmission. The fact that none of the heifers on 17 of the re-sampled farms seroconverted to BVDV was most likely due to the death or removal of PI animals before the first sampling event rather than very low BVDV transmission rates for co-mingled PI animals. Even under extensive beef farming conditions in New Zealand, we believe that the duration between sampling events (127 to 177 days; see Additional file 5) was sufficient for susceptible heifers to have effective contacts with PI animals if PI animals were actually present in the herd. In future studies with more available resources, it would be useful to track the serological status of replacement heifers from an earlier age and perform whole herd BVDV testing to determine the presence of PI animals as this would allow more accurate estimation of $\beta_{P}$.

Different values of $\beta_{P}$ have been suggested in the literature, but cannot be directly compared given that each group used different modelling structures and assumptions. However, the values can be indirectly compared by estimating the cumulative numbers of infected animals after 1 year of a PI animal being introduced to 100 susceptible animals in a closed herd while ignoring transmission by TI animals [10]. Using the previously reported $\beta_{P}$ values, the cumulative numbers of infected animals ranged from approximately 30 [6, 42] to 100 [5, 48, 49] with some intermediate estimations of 60 [42] and 84 [7]. When applying the same setting to $\beta_{P}$ values estimated in this study, it was equivalent to seroconvert 33 susceptible animals (mode) with a range between 10 and 71 animals (95% HPD interval). Our estimates are somewhat lower than those based on the previously reported $\beta_{P}$ values, indicating that using previous $\beta_{P}$ values to extensively grazed New Zealand beef farms may overestimate the transmission of BVDV. Given the fact that previous $\beta_{P}$ values were estimated based on either expert opinion or data from dairy herds, our estimate of $\beta_{P}$ would be more suitable for modelling BVDV in the pasture-based beef industry.

In addition to estimating $\beta_{P}$ values, we also discovered several interesting features of BVDV epidemiology from this study. First, the most probable proportion of introduced PI animals was estimated between 3 and 16%, which varied between farms. An anecdotal report showed that the prevalence of PI animals within a cattle herd in New Zealand varied between 2 and 12% [50], and so our estimate of proportion of introduced PI animals was in accordance with the previously reported range. Second, by considering the management feature, such as breeding and calving events, of replacement heifer herds, we were able to infer BVDV transmissions from introduced PI animals as well as any new born PI calves. In particular, Farms 1, 3, 6, and 7 showed that the estimated day of PI animals being introduced was during or immediately after the first calving period. This indicates that the introduced PI animals were actually new born PI calves either from heifers infected during early-mid pregnancy or potentially other purchased pregnant replacement heifers carrying a PI calf (Trojan dam). On the other hand, the most probable day of PI animals being introduced for Farms 2 and 9 was during the first breeding period, possibly indicating young PI replacement heifers were purchased for breeding. Unfortunately, we did not have detailed information on each farm’s purchasing history to confirm our speculations. However, these results still highlight that the ABC-SMC algorithm is a useful tool to infer unknown BVDV-related parameters, such as the timing and type of BVDV introductions, even with a limited amount of data.

In our simulation model, $\beta_{T}$ was assumed to be the product of $\beta_{P}$ and a proportion (0.05) that quantifies the reduced chance of the effective contacts with TI animals compared to PI animals, given that TI animals shed lower quantities of virus and likely require longer periods of time and closer contacts to cause transmission. Although we relied on other studies to calibrate this proportion, our preliminary investigation indicated that the proportion would have no marked impact on the estimated $\beta_{P}$ values, further implying that the uncertainty about $\beta_{T}$ could be negligible for estimating $\beta_{P}$. We also conducted a sensitivity analysis using an uniform and normal prior distribution of $\rho_{i}$. The sensitivity analysis showed that the posterior distributions of some parameters were highly affected by the prior distribution of $\rho_{i}$ (see Additional file 7). It indicates that some parameters, especially $\rho_{i}$, were not identifiable under the current model setting, possibly due to the lack of data and/or complex model structure, and the accurate estimation of parameter values in this study was generally dependent upon the precise information of $\uppi\left( {\rho_{i} } \right)$. Although we argue that our estimated parameter values were valid considering that the prior of $\rho_{i}$ in this study well-matched to the reported prevalence of new-born PI calves under New Zealand farming context [50], more observations—the number of tests as well as the sample size of each test—per farm should be warranted in the future study to mitigate the non-identifiability issue.

We consider several potential limitations to the study methods that could influence the accuracy of our estimates for $\beta_{P}$. First, the ABC-SMC algorithm was applied to data on only two sequential BVDV antibody ELISA tests for the subset of sampled animals in each herd. Even though we only accepted the parameters which were able to simulate data that closely matched the observed test results while adjusting for the test sensitivity and specificity, two sampling events may be regarded as insufficient. A lack of enough data points as well as errors introduced by the random sampling of heifers in this study might have increased the variability of the posterior distribution of not only $\beta_{P}$ but also other parameters [51]. More precise estimation could be achieved by conducting future longitudinal studies that sample more animals at more frequent intervals and test individual animals to confirm the presence and identity of PI animals. However, the logistical challenges of conducting sampling on extensively grazed beef herds will still remain as an obstacle, particularly since beef farmers are often reluctant to yard dams with calves at foot due to the risk of injury to calves [18]. Second, in our simulation model, we ignored any virus introduction from different management groups within the same farm or from other external sources (e.g. co-grazed sheep), which may have led to an overestimation of $\beta_{P}$ values. However, considering that tested heifers were grazed extensively, it is less likely that the heifers had frequent effective contacts with animals from different herds. Also, our current research about BVDV transmission between co-grazed cattle and sheep in New Zealand beef farms suggests that the transmission between two species is a rare event (unpublished). Finally, we assumed that PI animals were introduced at only one time point. However, based on unpublished observations from the national animal movement data in New Zealand and data from the management surveys completed by farmers, it is unlikely that many beef farmers are purchasing replacement heifers at multiple time points. The most likely alternate source of PI animals is through the birth of PI calves from susceptible animals that were exposed to BVDV during pregnancy. Therefore, we believe that the posterior distribution of $\beta_{P}$ in this study provides valid estimates of the most likely values.

Overall, the study findings have important clinical implications for the control of BVDV especially since the estimated $\beta_{P}$ values indicated that BVDV transmission is likely to be slow in extensively grazed beef herds. A similar finding was reported in a New Zealand cattle farm by Thompson [46], who observed that approximately 20% of animals remained susceptible to BVDV even after being directly co-grazed with PI animals for up to 600 days. These findings strongly suggest that using PI animals as a *“natural”* BVDV vaccination source (i.e. purposely exposing PI animals to susceptible animals to replicate the protective effect of BVDV vaccination) is likely to fail in the beef herds. On the contrary, this practice has a high risk of generating new PI calves in the next calving season with the risk of causing a BVDV outbreak in such extensively grazed beef cattle farms. Under these circumstances, effective prevention of BVDV should be based on test-and-cull of identified PI animals, vaccination of susceptible animals, and improvement of farm biosecurity [4].

## Supplementary information

**Additional file 1.** A copy of survey administered to New Zealand beef breeding farmers.

**Additional file 2.** Duration of protection against BVDV infection via maternal antibody.

**Additional file 3.** Detailed algorithm of approximate Bayesian computation-sequential Monte Carlo (ABC-SMC).

**Additional file 4.** General sensitivity analysis of model behaviour.

**Additional file 5.** Number of sampled and test-positive heifers at each sampling, herd size, breeding period, and day of both sampling events for each 9 New Zealand beef breeding farms.

**Additional file 6.** Evolution of parameter values and Inspection of estimated parameters.

**Additional file 7.** Sensitivity analysis.

## Data Availability

All data generated or analysed during this study are included in an additional file (see Additional file 5) and scripts are available at https://bit.ly/2sWibf5.

## Abbreviations

BVDV: bovine viral diarrhoea virus
ABC: approximate Bayesian computation
SMC: sequential Monte Carlo
PI: persistently infected
TI: transiently infected
HPD: highest posterior density
