# Supplementary material for: Estimation of the within-herd transmission rates of bovine viral diarrhoea virus in extensively grazed beef cattle herds
Source: Vet Res. 2019 Nov 29;50:103. doi: 10.1186/s13567-019-0723-2 (PMC6884759; doi:10.1186/s13567-019-0723-2)
Supplement: Supplementary file 1 — Additional file 1. A copy of survey administered to New Zealand beef breeding farmers. [file 13567_2019_723_MOESM1_ESM.pdf]

BVD FREE NEW ZEALAND

Building grassroots capacity to eradicate bovine viral diarrhoea virus

BVD Management Survey for Beef Herds

Dear Farmer,

Bovine viral diarrhoea virus (BVD) is a common infectious disease of cattle that costs New Zealand farmers more than **$150 million per year** from direct production losses in the **15% of dairy herds** and **65% of beef herds** that are actively infected with the virus. BVD even has significant economic impacts on virus-free herds due to the high ongoing costs of testing and vaccinating cattle to prevent future disease outbreaks.

Several European countries have already launched successful national BVD eradication programmes and we strongly believe that eradicating BVD from New Zealand would be technically feasible and highly profitable for the cattle industries. However, we need a lot more information on how the virus spreads between farms and how much it currently affects herd performance to prepare a sound business case around the different BVD management options (including voluntary control, phased-in mandatory control, and fast-track eradication).

- **Why are we running this survey?**
  Every farm in New Zealand has a unique management style and different risk factors for BVD. We specifically want to know how BVD affects your herd and what control measures would be practical for you to put into place. This information will allow us to design and test different national BVD control programmes using an innovative computer simulation model to find the approach that will have the greatest financial benefit at the lowest cost to your farm business.

- **What will the survey involve?**
  This BVD Management Survey should take around 30 minutes to complete and has five sections that will ask you questions about your (1) Contact Details and Background, (2) BVD Testing History, (3) Farm Management Practices, (4) BVD Biosecurity Risks, and (5) Opinions Towards National Control. The survey may be completed on paper and either posted or scanned and e-mailed to:

Carolyn Gates (BVD Free New Zealand)
Massey University
Institute of Veterinary, Animal and Biomedical Science (IVABS)
Private Bag 11-222 Palmerston North, New Zealand 4442
Email: c.gates@massey.ac.nz

  We will also shortly be offering the option of completing the survey online. Please visit the project website (www.bvdfree.org.nz) for more details.

- **How will we use the information?**
  Any information you provide will be treated as strictly confidential and used only for the purpose of understanding how we can better manage BVD in New Zealand. If you have any questions or concerns about participating in the project, please do not hesitate to contact the Project Manager (Carolyn Gates) at c.gates@massey.ac.nz or 06 951 8140.

This Sustainable Farming Fund research project is an exciting opportunity to change how we control infectious diseases in New Zealand cattle industry and we look forward to working with you over the next three years.

Sincerely,

Dr. Carolyn Gates
Project Manager

This research programme was made possible through the generous financial support of Sustainable Farming Fund, AGMARDT, Massey University, MSD Animal Health, and Zoetis. We are grateful to our partners at the National BVD Steering Committee, MPI, DairyNZ, Beef&LambNZ, OSPRI, Gribbles Pathology, NZVP/IDEXX, LIC, SVS Laboratories, ThermoFisher, NZVA, RMPP, and private veterinary practices.

BVD Management Survey for Beef Herds

Visit www.bvdfree.org.nz today to find out more about the research project and how you can get behind BVD control in New Zealand.

Section 1: Contact Details and Background Information

Farmer Contact Details	
Name	
Street Address	
Town	
Postal code	
E-mail	
Phone	
Veterinary Clinic	
Primary Veterinarian	

Background Information	
Are you primarily responsible for making management decisions on the farm?	☐ Yes ☐ No
Gender:	☐ Male ☐Female<br>☐ Prefer Not to Answer
Age:	☐ Under 25 ☐25 to 34<br>☐ 35 to 44 ☐45 to 54<br>☐ 55 to 64 ☐Over 65<br>☐ Prefer Not to Answer
Number of years farming beef cattle:	__________
Highest education level:	☐ High school ☐ Undergraduate<br>☐ Masters ☐Doctoral
Ethnic background:	☐ Maori ☐ New Zealand European<br>☐ Other: ______________________<br>☐ Prefer Not to Answer
Are you willing to be contacted to participate in future research studies?	☐ Yes ☐No

BVD Management Survey for Beef Herds

Visit www.bvdfree.org.nz today to find out more about the research project and how you can get behind BVD control in New Zealand.

Section 2: BVD Testing History

Previous Testing

Do you believe your herd currently has an active BVD infection?			☐ Yes ☐ No ☐ Unsure
Has your herd been screened for BVD within the last 5 years to determine exposure status?			☐ Yes ☐ No ☐ Unknown
**If No:**	What was the main reason for not performing testing (select ONE)?		☐ Unaware of BVD<br>☐ Too expensive<br>☐ Low perceived impact of BVD<br>☐ No intention to control<br>☐ Other: ____________________
**If Yes:**	How often do you screen for BVD?		☐ Annually ☐ Other: __________________
	When was the last screening test?		_____ / __________ (mm/yyyy)
	What screening test(s) were used?		☐ Sampling 10-15 youngstock to check for antibodies<br>☐ Screening all calves in the herd for virus<br>☐ Screening all animals in the herd for virus<br>☐ Other __________________________
	What was the result?		☐ Negative ☐ Positive
	**If Positive:**	Was follow up testing performed to identify individual PI animals?	☐ Yes ☐ No
Have you had a known persistently infected animal(s) in the herd within last 5 years?			☐ Yes ☐ No<br>☐ Unknown (testing not done)
**If Yes:**	When was the last PI identified?		_____ / __________ (mm/yyyy)
	What was the outcome for PI animals?		☐ Culled<br>☐ Sold<br>☐ Remained in herd<br>☐ Other: ______________________

BVD Management Survey for Beef Herds

Visit www.bvdfree.org.nz today to find out more about the research project and how you can get behind BVD control in New Zealand.

Section 3: Farm Management Information

Farm Location

Please provide the NAIT numbers for all locations **in your ownership** where your cattle are grazed

________________________ ________________________ ________________________

________________________ ________________________ ________________________

Please provide the NAIT numbers for all locations **NOT in your ownership** where your cattle are grazed-off (i.e. heifer rearers or off-site grazing)

________________________ ________________________ ________________________

________________________ ________________________ ________________________

What type of livestock operations are located on your farm? (Check all that apply)	☐ Beef breeding<br>☐ Beef finishing<br>☐ Dairy milking<br>☐ Sheep<br>☐ Dairy grazing<br>☐ Deer<br>☐ Other ____________________
Please provide the size of effective grazing area of your enterprise.	__________ hectares

Herd Demographics

Please answer the following questions related to the 2016/17 season (1 July 2016 to 30 June 2017).

What was the total number of cattle on the farm at the planned start of calving?	__________
Please provide the total number of cattle on the farm by management type at the planned start of calving.	☐ Breeding cows ___________<br>☐ Breeding bulls ___________<br>☐ Store cattle (weaning to slaughter) ___________<br>☐ Replacement heifers (Rising 1 year olds) ___________<br>☐ Replacement heifers (Rising 2 year olds) ___________<br>☐ Other ___________

BVD Management Survey for Beef Herds

Visit www.bvdfree.org.nz today to find out more about the research project and how you can get behind BVD control in New Zealand.

Please describe how your herd is managed in different mobs (i.e. which cattle are grazed together at different times of year).

** This is to help us understand how BVD might spread within your herd and what would be the most cost-effective strategy to test your herd for BVD. **

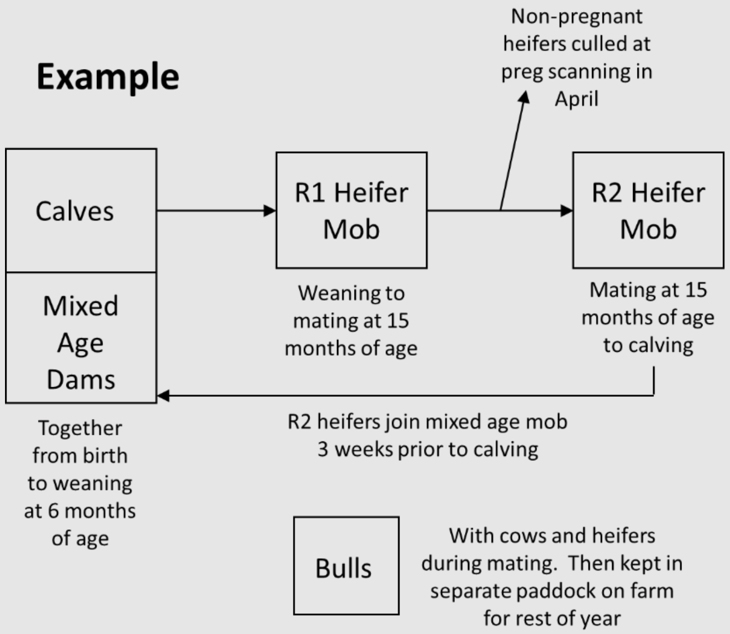

BVD Management Survey for Beef Herds

Visit www.bvdfree.org.nz today to find out more about the research project and how you can get behind BVD control in New Zealand.

What approximate dates during the year are beef cattle yarded for routine management events?
** This is to help us understand when BVD testing and/or vaccination could be integrated into your routine management calendar. **

For Mob, indicate whether they are:
Calves (C) , R1 Heifers (R1), R2 Heifers (R2), Mixed Age Cows (MA), or Bulls (B)

Events could include drenching, vaccination, scanning, etc

☐ Date ____/______ / _________ Mob: _____ Event: ____
☐ Date ____/______ / _________ Mob: _____ Event: ____
☐ Date ____/______ / _________ Mob: _____ Event: ____
☐ Date ____/______ / _________ Mob: _____ Event: ____

☐ Date ____/______ / _________ Mob: _____ Event: ____
☐ Date ____/______ / _________ Mob: _____ Event: ____
☐ Date ____/______ / _________ Mob: _____ Event: ____
☐ Date ____/______ / _________ Mob: _____ Event: ____

☐ Date ____/______ / _________ Mob: _____ Event: ____
☐ Date ____/______ / _________ Mob: _____ Event: ____
☐ Date ____/______ / _________ Mob: _____ Event: ____
☐ Date ____/______ / _________ Mob: _____ Event: ____

☐ Date ____/______ / _________ Mob: _____ Event: ____
☐ Date ____/______ / _________ Mob: _____ Event: ____
☐ Date ____/______ / _________ Mob: _____ Event: ____
☐ Date ____/______ / _________ Mob: _____ Event: ____

☐ Date ____/______ / _________ Mob: _____ Event: ____
☐ Date ____/______ / _________ Mob: _____ Event: ____
☐ Date ____/______ / _________ Mob: _____ Event: ____
☐ Date ____/______ / _________ Mob: _____ Event: ____

BVD Management Survey for Beef Herds

Visit www.bvdfree.org.nz today to find out more about the research project and how you can get behind BVD control in New Zealand.

Reproductive Management and Performance

Please answer the following questions related to **calving** during the 2016/17 season (1 July 2016 to 30 June 2017). *This is to help us understand the impact of BVD on herd performance.*

When did calving start in the 2016 season?		_____ / _____ / __________
How many total calves were weaned?		_________
How many total calves were born?		_________
**If you know:**	What was the approximate calving distribution (% of animals calving in each 3 week period)?	Weeks 1 to 3 ______ Weeks 7 to 9 ______<br>Weeks 4 to 6 ______ Weeks 10 to 12 ______
Were any of the calves stunted or born with birth defects?		☐ Yes ☐ No
**If Yes:**	Please provide additional details around the number of animals and clinical signs	
Did you measure the growth rate of calves from birth to weaning?		☐ Yes ☐ No
**If Yes:**	What was the average daily gain (kg/day)?	_________

Please answer the following questions related to **mating** during the 2016/17 season (1 July 2016 to 30 June 2017).

When did mating start in the 2016 season?		Cows _____ / _____ / _________<br>Heifers _____ / _____ / __________
How many bulls were used?		_________
How long was the mating period (weeks)?		Cows __________ Heifers __________
How many total females were mated?		Cows __________ Heifers __________
Was pregnancy scanning performed after the end of mating?		☐ Yes ☐ No
**If Yes:**	What was the empty rate (% of animals not pregnant)?	Cows __________ Heifers __________
How many breeding animals were culled?		Cows __________ Heifers __________

BVD Management Survey for Beef Herds

Visit www.bvdfree.org.nz today to find out more about the research project and how you can get behind BVD control in New Zealand.

Veterinary Costs and Information Sources

Please answer the following questions related to the 2016/17 season (1 July 2016 to 30 June 2017).

This is to help us understand the impact of BVD on other animal health issues.

How many cases of scours did you treat?	______________
How much on did you spend for non-routine veterinary care to treat scours and/or respiratory disease? Please exclude the cost of routine vaccination or drenching	NZD _______________

What are the top 3 animal health concerns for your beef operation?

1. ____
____
2. ____
____
3. ____
____

On a scale of 1 (no knowledge) to 10 (expert), how would you rate your knowledge of BVD?	
Where do you currently receive most of your information about BVD?	☐ Veterinarian<br>☐ Industry Magazines / Publications<br>☐ BVD Steering Committee Website<br>☐ Other farmers<br>☐ Other<br>_____________________________________

What could be done to improve how information about BVD is communicated?

BVD Management Survey for Beef Herds

Visit www.bvdfree.org.nz today to find out more about the research project and how you can get behind BVD control in New Zealand.

Section 4: Biosecurity Information

Purchased Cattle

Please answer the following questions related to the 2016/17 season (1 July 2016 to 30 June 2017).

Did you purchase any cattle during this time period (including breeding bulls)? ☐ Yes ☐ No

If Yes: Please provide the approximate number purchased by management type and indicate how many of these were tested for BVD and/or vaccinated for BVD before entering your farm.

	Number purchased	Number of these BVD tested	Number of these BVD vaccinated
Breeding cows			
Breeding bulls			
Calves (pre-weaning)			
Store/finishing cattle (weaning to slaughter)			
Replacement heifers (Rising 1 year olds)			
Replacement heifers (Rising 2 year olds)			

How often were the purchased cattle isolated before being mixed with the herd?

☐ Never ☐ Rarely ☐ Sometimes ☐ Often ☐ Always

If you isolate the purchased cattle: How long were the purchased cattle isolated before being mixed? __________ days

How often did you ask about the BVD disease status of the source herd(s)?

☐ Never ☐ Rarely ☐ Sometimes ☐ Often ☐ Always

How often did you ask about the BVD vaccination status of the source herd(s)?

☐ Never ☐ Rarely ☐ Sometimes ☐ Often ☐ Always

What factors influenced your decisions around BVD biosecurity for purchased cattle?

BVD Management Survey for Beef Herds

Visit www.bvdfree.org.nz today to find out more about the research project and how you can get behind BVD control in New Zealand.

Sold Cattle

Please answer the following questions related to the 2016/17 season (1 July 2016 to 30 June 2017).

Did you sell any cattle to other farms during this time period?	☐ Yes ☐ No

If Yes: Please provide the number of each type of animal sold

☐ Breeding cows ___________

☐ Breeding bulls ___________

☐ Calves (pre-weaning) ___________

☐ Store/finishing cattle (weaning to slaughter) ___________

☐ Replacement heifers (Rising 1 year olds) ___________

☐ Replacement heifers (Rising 2 year olds) ___________

How often did the buyer(s) ask about the BVD disease status of your herd?

☐ Never ☐ Rarely ☐ Sometimes ☐ Often ☐ Always

How often did the buyer(s) ask about the BVD vaccination status of your herd?

☐ Never ☐ Rarely ☐ Sometimes ☐ Often ☐ Always

Neighbouring Farms

Do you have any neighbour(s) who graze cattle on pastures with shared fenceline boundaries to your cattle?		☐ Yes ☐ No
**If Yes:**	Please provide the number of properties with cattle sharing boundaries with your farm.	___________
	Do any of the fenceline boundaries permits direct nose-to-nose contact with neighbouring cattle?	☐ Yes ☐ No
	What is the BVD status of the neighbouring farms?	☐ All negative<br>☐ All positive<br>☐ Mixed positive and negative<br>☐ Don't know
Do you share yards, pasture, pond or other water sources with your neighbour(s)?		☐ Yes ☐ No
Do you share equipment, such as dehorners or trailers, with your neighbour(s)?		☐ Yes ☐ No

BVD Management Survey for Beef Herds

Visit www.bvdfree.org.nz today to find out more about the research project and how you can get behind BVD control in New Zealand.

Off-Site Grazing and Movements

Please answer the following questions related to the 2016/17 season (1 July 2016 to 30 June 2017).

Were any of your cattle moved off-site for grazing?			☐ Yes ☐ No
**If Yes:**	Approximately how long were the animals off-site?		__________ (weeks)
	Were the animals co-grazed with other herds?		☐ Yes ☐ No
	**If Yes:**	Were the other herds known to be free from BVD?	☐ Yes ☐ No ☐ Did not ask
	Were any of the animals grazed off-site pregnant at any time during that period?		☐ Yes ☐ No
	**If Yes:**	Were these animals vaccinated prior to conception?	☐ Yes ☐ No
		Were the calves from these animals tested for BVD?	☐ Yes ☐ No
		Were the animals isolated on their return?	☐ Yes ☐ No
Were any cattle from other herds moved onto your site for grazing?			☐ Yes ☐ No
**If Yes:**	How many herds did the cattle originate from?		__________
	Were these cattle co-mingled with your stock?		☐ Yes ☐ No ☐ Did not ask
	Were these cattle known to be free from BVD?		☐ Yes ☐ No ☐ Did not ask
	Were these cattle vaccinated against BVD?		☐Yes ☐No ☐Did not ask
Were other cattle moved off farm and returned for any of the following reasons?			☐ Attending show<br>☐ Breeding elsewhere<br>☐ Veterinary treatment<br>☐ Other ___________________________

BVD Management Survey for Beef Herds

Visit www.bvdfree.org.nz today to find out more about the research project and how you can get behind BVD control in New Zealand.

BVD Vaccination

Please answer the following questions related to the 2016/17 season (1 July 2016 to 30 June 2017).

Did you vaccinate any of your cattle for BVD?		☐ Yes ☐ No
**If No:**	What was your primary reason for not vaccinating?	☐ BVD not present in the herd<br>☐ Vaccination too expensive or impractical<br>☐ BVD present, but not impacting herd performance<br>☐ BVD present, but no intention to control<br>☐ Other ______________________________
**If Yes:**	What vaccine product was used?	☐ OneShot BVD ☐ Bovilis ☐ Bovi-shield Gold<br>☐ Other ______________________________
	Which groups of animals were vaccinated for BVD?	☐ Breeding cows<br>☐ Breeding bulls<br>☐ Calves (pre-weaning)<br>☐ Store/finishing cattle (weaning to slaughter)<br>☐ Replacement heifers (Rising 1 year olds)<br>☐ Replacement heifers (Rising 2 year olds)
	Approximately what dates were the vaccines given?	

Contact with People

How often do you see uninvited visitors/trampers passing through your property?

☐ Never ☐ Rarely ☐ Sometimes ☐ Often ☐ Always

How often do people (e.g. vets, calf dehorners, or scanners) who work with animals wash their boots and other equipment before or after making contact with your cattle?

☐ Never ☐ Rarely ☐ Sometimes ☐ Often ☐ Always

How many visits from the following type of personnel occur on average every month?

	Jan	Feb	Mar	Apr	May	Jun	Jul	Aug	Sep	Oct	Nov	Dec
Veterinarians	____	____	____	____	____	____	____	____	____	____	____	____
Livestock transport vehicles	____	____	____	____	____	____	____	____	____	____	____	____

Question continued on next page

BVD Management Survey for Beef Herds

Visit www.bvdfree.org.nz today to find out more about the research project and how you can get behind BVD control in New Zealand.

	Jan	Feb	Mar	Apr	May	Jun	Jul	Aug	Sep	Oct	Nov	Dec
Farm advisors	____	____	____	____	____	____	____	____	____	____	____	____
Stock agents	____	____	____	____	____	____	____	____	____	____	____	____

Contact with Sheep

Please answer the following questions related to the 2016/17 season (1 July 2016 to 30 June 2017).

Please provide the average number of each sheep type on your farm.		☐ No sheep present<br>☐ Mixed age ewes ___________<br>☐Two-tooth ewes ___________<br>☐Ewe lamb/hogget ___________<br>☐ Mixed age rams ___________
How many ewes were mated in the 2016 season?		__________
Please provide information on the following production parameters (if available).		Scanning percentage ___________<br>Lambing percentage ___________<br>Weaning percentage ___________
Were any of your cattle directly co-grazed with a sheep flock?		☐ No<br>☐ Yes - in the same paddock at the same time<br>☐ Yes - in adjacent paddocks with possible contact through fenceline<br>☐ Yes - on the same paddock, but at different times
**If Yes:**	Which management groups were co-grazed with sheep?	☐ Breeding cows<br>☐ Breeding bulls<br>☐ Calves (pre-weaning)<br>☐ Store/finishing cattle (weaning to slaughter)<br>☐ Replacement heifers (Rising 1 year olds)<br>☐ Replacement heifers (Rising 2 year olds)
Has your sheep flock ever been diagnosed with Hairy shaker (Border) disease?		☐ Yes ☐ No ☐ Unsure

BVD Management Survey for Beef Herds

Visit www.bvdfree.org.nz today to find out more about the research project and how you can get behind BVD control in New Zealand.

Section 5: Opinions Towards National Disease Control

Are you aware of the national BVD control programmes that countries in Europe have implemented?	☐ Yes ☐ No
Do you believe it is possible to eradicate BVD from New Zealand?	☐ Yes ☐ No ☐ Unsure
On a scale of 1 (least supportive) to 10 (most supportive), how strongly do you support having a coordinated national BVD eradication programme in New Zealand?	___________

Comments

Which of the following types of coordinated national BVD control programme would you most support?	☐ **Voluntary** – Decision to control BVD is left entirely to individual farmers<br>☐ **Phased** – Level of BVD control is progressively increased from voluntary to compulsory over several years<br>☐ **Compulsory** – BVD control is legislated by the government from the start

Comments

Who should decide what approach to national BVD eradication New Zealand should take?	☐ National BVD Steering Committee<br>☐ Farmers (by vote)<br>☐ Industry (DairyNZ and Beef&LambNZ)<br>☐ Other: ______________________________

Comments

BVD Management Survey for Beef Herds

Visit www.bvdfree.org.nz today to find out more about the research project and how you can get behind BVD control in New Zealand.

Which of the following BVD control measures would you consider **voluntarily** implementing on your farm?	☐ Purchasing BVD free or vaccinated animals only<br>☐ Isolation of cows brought in to your farm<br>☐ Double fencing on your farm boundary<br>☐ Testing calves of animals moved off-site during pregnancy<br>☐ Testing all replacement calves<br>☐ Testing all replacement bulls and heifers<br>☐ Only co-grazing cattle with herds that are free from BVD<br>☐ Vaccinating at-risk stock against BVD
**Comments**	
Which of the following BVD control measures would you support as part of a **mandated** national BVD eradication programme?	☐ Screening annually to establish BVD status<br>☐ Requiring herds to declare BVD status at the time of sale<br>☐ Restricting movements of animals shedding BVD virus<br>☐ Establishing a national database to record herd BVD status<br>☐ Mandating that BVD positive herds take appropriate measures to control disease
**Comments**	

BVD Management Survey for Beef Herds

Visit www.bvdfree.org.nz today to find out more about the research project and how you can get behind BVD control in New Zealand.

What do you see as the biggest **benefits** to eradicating BVD from New Zealand?

What do you see as the biggest **challenges** to eradicating BVD from New Zealand?

What features could be built into an eradication programme to increase success?

**Additional Comments**

Thank you for your time!
