# Supplementary material for: Estimation of the within-herd transmission rates of bovine viral diarrhoea virus in extensively grazed beef cattle herds
Source: Vet Res. 2019 Nov 29;50:103. doi: 10.1186/s13567-019-0723-2 (PMC6884759; doi:10.1186/s13567-019-0723-2)
Supplement: Supplementary file 2 — Additional file 2. Duration of protection against BVDV infection via maternal antibody. [file 13567_2019_723_MOESM2_ESM.docx]

## Additional file 2 Duration of protection against BVDV infection via maternal antibody

We estimated that the duration of protection against BVDV infection via maternal antibody (Ab) using the reported data of other literature. First, we assumed that the minimum Ab titre required for protection against BVDV infection was 1:8 [22, 24]. We then extracted the reported data of maternal Ab titre of individual calves over time from two studies [37, 38]. For the extraction, only the data of animals; (1) with sufficient (>1:8) initial Ab titre, and (2) without any evidence of BVDV infection before the titre dropped to an undetectable level (1:1). Using the extracted data, the decay rate of maternal Ab in the binary logarithm (${log}_{2}$) scale was estimated by using generalised linear mixed model with the individual calf id as a random effect. The mean duration of protection against BVDV infection was estimated by calculating the age with maternal Ab titre matching to 3 ($\log_{2} 8$) based on the estimated decay rate and initial Ab titre (i.e. intercept). To adjust the variation of the titre between calves, we assumed that the duration of protection followed normal distribution, and the standard deviation of the duration was estimated by dividing the standard deviation of the random effect by the coefficient of age.

**Table S1. Result of generalised linear mixed model on BVDV maternal antibody titre in binary logarithm scale.**

| Variable | Coefficient | Standard error | *P* value |
| --- | --- | --- | --- |
| Random effect (Calves) | N/A | 1.305 * | < 0.0001 ** |
| Intercept | 9.546 | 0.493 | < 0.0001 |
| Age | -0.042 | 0.002 | < 0.0001 |

Key: N/A, Not available.

* Standard deviation of the random effect

** *P* value of log-likelihood test

The estimated decay rate of maternal Ab was -0.042 which was in accordance with previously estimated values in other studies (Table S1) [23, 25]. The estimated initial maternal Ab titre was 9.546 (in the binary logarithm scale), and the mean duration of protection against BVDV infection was 154.8 days. The standard deviation of the random effect was 1.305, and the standard deviation of the duration of protection was 30.8 days. Therefore, the mean and standard deviation of the duration of protection against BVDV infection via maternal Ab was approximated to 155 and 31 days, respectively (Figure S1).


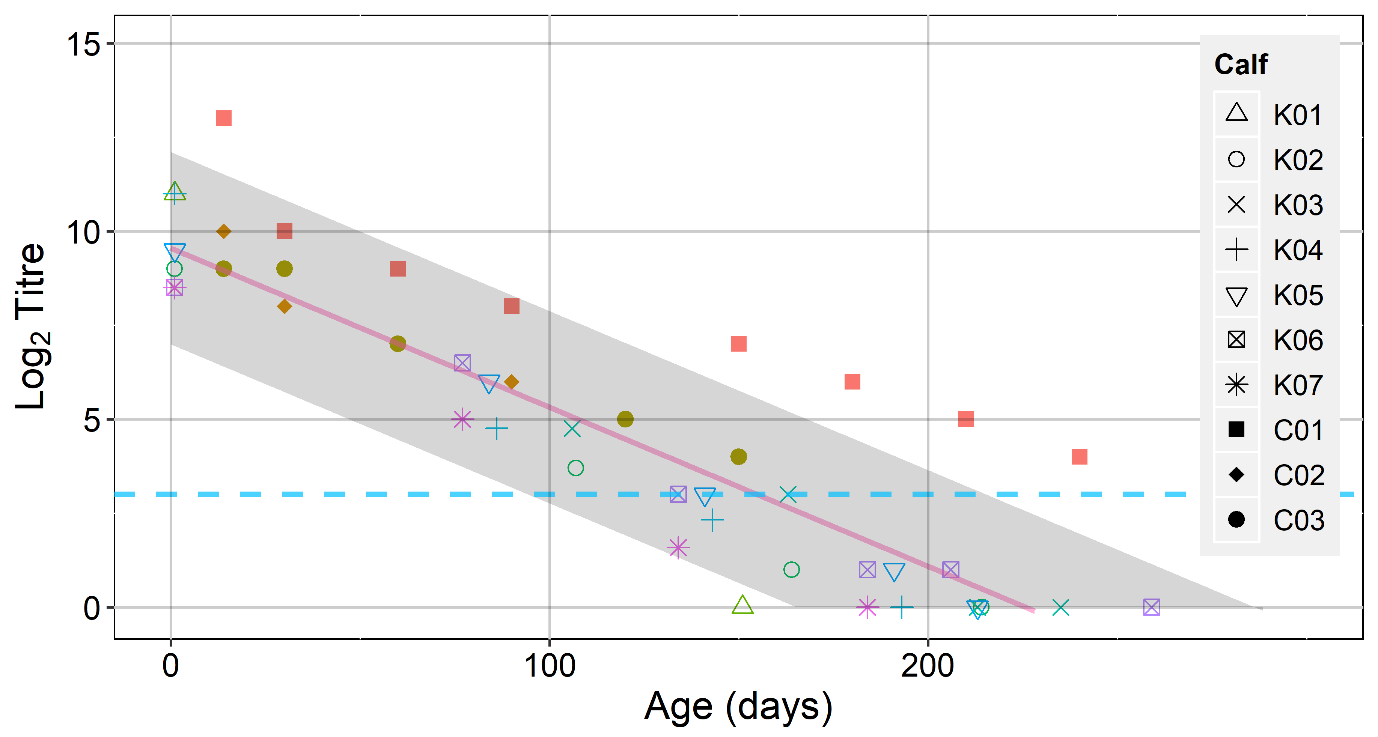


**Figure S1. Decay rate of BVDV maternal antibody titre in binary logarithm scale.** Blue dashed horizontal line indicates the minimum antibody titre required for protection against BVDV infection.
