# Supplementary material for: Estimation of the within-herd transmission rates of bovine viral diarrhoea virus in extensively grazed beef cattle herds
Source: Vet Res. 2019 Nov 29;50:103. doi: 10.1186/s13567-019-0723-2 (PMC6884759; doi:10.1186/s13567-019-0723-2)
Supplement: Supplementary file 3 — Additional file 3. Detailed algorithm of approximate Bayesian computation-sequential Monte Carlo (ABC-SMC). [file 13567_2019_723_MOESM3_ESM.docx]

## Additional file 3 Detailed algorithm of approximate Bayesian computation-serial Monte Carlo (ABC-SMC).

To estimate unknown parameters for each of 9 beef herds, we implemented ABC-SMC algorithm in the following order. Since we estimated multiple parameters at the same time, *θ* below is the vector of parameters.

1. Set *t* = 1, where *t* is the SMC sequence indicator. Initialise the threshold values for the first round (t = 1), $\varepsilon_{1}(1)$ and $\varepsilon_{2}(1)$, by;
   1. Running the simulation model 2000 times without rejecting any particles, and estimating the distances ($D_{k}$) of summary statistics as;

$$D_{k}=\sqrt{\sum_{i=1}^{n} {{(T_{i}(+)}_{obs}^{k}-{T_{i}\left( + \right)}_{sim}^{k})}^{2}}$$

where *n* is the number of herds, ${T_{i}(+)}_{obs}^{1}$ and ${T_{i}(+)}_{sim}^{1}$ are the observed and simulated number of test positive heifers in the first sampling round for herd *i*, respectively, ${T_{i}(+)}_{obs}^{2}$ and ${T_{i}(+)}_{sim}^{2}$ are the observed and simulated number of seroconverted heifers in the second sampling round for herd *i*, respectively.

- 1. Setting $\varepsilon_{1}(1)$ and $\varepsilon_{2}(1)$ as the median values of $D_{1}$ and $D_{2}$, respectively.

1. Set *i* = 1, where *i* is the particle indicator.
2. Generate a particle of parameter set, *θ*, by;
   1. If *t* = 1, sample $\theta^{**}$ from $\pi(\theta)$, where $\pi(\theta)$ is the prior distributions of *θ*.
   2. If *t* > 1, sample $\theta^{*}$ from the particles of previous sequence, {$\theta_{t-1}$}, with weights, {$w_{t-1}$}. Then perturb the particle $\theta^{**} \sim K(\theta|\theta^{*})$, where $K(\cdot)$ is a perturbation kernel. In this study, we used a component-wise Gaussian kernel with the variance as 0.68 times of the variance of particles in the previous SMC sequence. If the probability of $\pi(\theta^{**})$ equals 0, return to (3).
3. Run the simulation model with the generated particles and calculated $D_{k}$.
4. Accept $\theta^{**}$ as the particle $\theta_{t}^{i}$ if $D_{1}$ < $\varepsilon_{1}(t)$ and $D_{2}$ < $\varepsilon_{2}(t)$, otherwise return to (3).
5. Calculate weight for the particle, $w_{t}^{i}$, as;
   1. If *t* = 1, $w_{t}^{i}=1$,
   2. If *t* > 1, $w_{t}^{i}= {\pi(\theta_{t}^{i})}/{\sum_{j=1}^{N} w_{t-1}^{j}K(\theta_{t}^{i},\theta_{t-1}^{j})}$.
6. Set *i* = *i* + 1, and repeat (3) ~ (6) until *i* = 2004. It indicates that 2000 particles for each parameter were accepted for one SMC sequence.
7. Normalise the weights (divide the weight by the sum of weight) and calculate the effective sample size (ESS) as;

$$ESS=\frac{1}{\sum_{1}^{2000} {(\bar{w}^{i})}^{2}}$$

where $\bar{w}^{i}$ was normalised weight.

1. Calculate new threshold for the next sequence, $\varepsilon_{1}(t+1)$ and $\varepsilon_{2}(t+1)$ from the median values of $D_{1}$ and $D_{2}$, respectively, and set *t* = *t* + 1. Return to (1) until *t* = 15.

In total, we sampled approximately 97.2 million particles and ESS of any parameter during the whole sequence was between 795 and 2000.
