# Supplementary material for: Estimation of the within-herd transmission rates of bovine viral diarrhoea virus in extensively grazed beef cattle herds
Source: Vet Res. 2019 Nov 29;50:103. doi: 10.1186/s13567-019-0723-2 (PMC6884759; doi:10.1186/s13567-019-0723-2)
Supplement: Supplementary file 4 — Additional file 4. General sensitivity analysis of model behaviour. [file 13567_2019_723_MOESM4_ESM.docx]

## Additional file 4 General sensitivity analysis of model behaviour.

Since the simulation model in this study was newly developed, we investigated the model behaviour with respect to different parameter values. The sensitivity analysis was conducted using a complete factorial design on five unknown parameters; (1) within-herd BVDV transmission rate for PI animals ($\beta_{P}$), (2) the initial proportion of BVDV seropositive animals ($\mu$), (3) the proportion of introduced PI animals ($\rho$), (4) the day of PI animals being introduced ($\tau$), and (5) the proportional difference of $\beta_{T}$ compared to $\beta_{P}$. Five values (0.1, 0.3, 0.5, 0.7, and 0.9) were tested for all parameters except $\tau$, and the days equivalent to 10^th^, 30^th^, 50^th^, 70^th^, and 90^th^ percentile of the duration between the day of weaning (day 0) and the day of first sampling were used for $\tau$, resulting in testing 3125 different scenarios. Each scenario was simulated for 1000 times to adjust the variation caused by stochasticity. For each simulation, we stored the parameter values used and the number of seropositive heifers at the first and second sampling events. Once whole simulation was over, a dataset of 3 125 000 observations was created, and the numbers of seropositive heifers were separately analysed using multivariable Poisson regression with the parameters as explanatory variables. For the simplicity, the analysis was conducted under the management features of Farm 1 only.

**Table S2. Multivariable Poisson regression on the number of seropositive heifers at two sampling events.**

|  | Number of seropositive heifers at; | | | | |
| --- | --- | --- | --- | --- | --- |
| Variable | First sampling | |  | Second sampling | |
|  | Odds ratio (95% CI) | *P*-value |  | Odds ratio (95% CI) | *P*-value |
| Intercept | 0.688 (0.687, 0.689) | < 0.0001 |  | 0.764 (0.763, 0.765) | < 0.0001 |
| $\beta_{P}$ | 1.050 (1.049, 1.052) | < 0.0001 |  | 1.025 (1.024, 1.026) | < 0.0001 |
| *μ* | 1.756 (1.753, 1.758) | < 0.0001 |  | 1.525 (1.523, 1.526) | < 0.0001 |
| *ρ* | 0.595 (0.594, 0.595) | < 0.0001 |  | 0.659 (0.658, 0.660) | < 0.0001 |
| *τ* | 1.000 (1.000, 1.000) | < 0.0001 |  | 1.000 (1.000, 1.000) | < 0.0001 |
| *K* | 1.014 (1.013, 1.015) | < 0.0001 |  | 1.005 (1.004, 1.006) | < 0.0001 |
| Key: CI, confidence interval; K, the proportional difference of $\beta_{T}$ compared to $\beta_{P}$. | | | | | |

Table above illustrates the results of multivariable Poisson regression models. Not surprisingly, the number of seropositive heifers at both the first and second sampling were the most sensitive to $\mu$, followed by $\rho$. Compared to $\mu$ or $\rho$, $\beta_{P}, \tau$ and the proportional difference of $\beta_{T}$ compared to $\beta_{P}$ had only a limited impact on the number of seropositive heifers in both sampling occasions. This result indicates that the posterior distribution of $\beta_{P}$ using ABC-SMC in this study is expected to be mainly affected by the values of $\mu$ and $\rho$ since the estimation of $\beta_{P}$ in ABC-SMC of this study depended on the number of seropositive heifers at the first sampling and the number of seroconverted heifers at the second sampling events.
