# Supplementary material for: Estimation of the within-herd transmission rates of bovine viral diarrhoea virus in extensively grazed beef cattle herds
Source: Vet Res. 2019 Nov 29;50:103. doi: 10.1186/s13567-019-0723-2 (PMC6884759; doi:10.1186/s13567-019-0723-2)
Supplement: Supplementary file 5 — Additional file 5. Number of sampled and test-positive heifers at each sampling, herd size, breeding period, and day of both sampling events for each 9 New Zealand beef breeding farms. [file 13567_2019_723_MOESM5_ESM.docx]

## Additional file 5 Number of sampled and test-positive heifers at each sampling, herd size, breeding period, and day of both sampling events for each 9 New Zealand beef breeding farms.

| Farm | Herd size | # Sampled | # 1^st^ positive | # 2^nd^ positive | Breeding period ^†^ | Day of 1^st^ sampling ^†^ | Day of 2^nd^ sampling ^†^ |
| --- | --- | --- | --- | --- | --- | --- | --- |
| 1 | 40 | 15 | 9 | 6 | 250 ~ 306 | 590 | 735 |
| 2 | 90 | 14 | 11 | 1 | 230 ~ 265 | 549 | 690 |
| 3 | 66 | 15 | 2 | 6 | 203 ~ 245 | 533 | 711 |
| 4 | 23 | 15 | 13 | 2 | 236 ~ 292 | 579 | 731 |
| 5 | 24 | 15 | 13 | 2 | 571 ~ 641 | 946 | 1,097 |
| 6 | 37 | 14 * | 7 | 4 | 250 ~ 292 | 625 | 752 |
| 7 | 20 | 15 | 3 | 9 | 590 ~ 646 | 950 | 1,097 |
| 8 | 20 | 15 | 12 | 3 | 236 ~ 320 | 597 | 736 |
| 9 | 29 | 15 | 14 | 1 | 240 ~ 261 | 640 | 794 |

* Numbers of sampled replacement heifers at the first round adjusting for the censored heifers at the second sampling round. Originally 15 heifers were sampled at the first round.

^†^ Day of each event was measured from day 0 which was the day of heifers being weaned.
