# Supplementary material for: Estimation of the within-herd transmission rates of bovine viral diarrhoea virus in extensively grazed beef cattle herds
Source: Vet Res. 2019 Nov 29;50:103. doi: 10.1186/s13567-019-0723-2 (PMC6884759; doi:10.1186/s13567-019-0723-2)
Supplement: Supplementary file 6 — Additional file 6. Evolution of parameter values and Inspection of estimated parameters. [file 13567_2019_723_MOESM6_ESM.docx]

## Additional file 6. Evolution of parameter values and Inspection of estimated parameters.


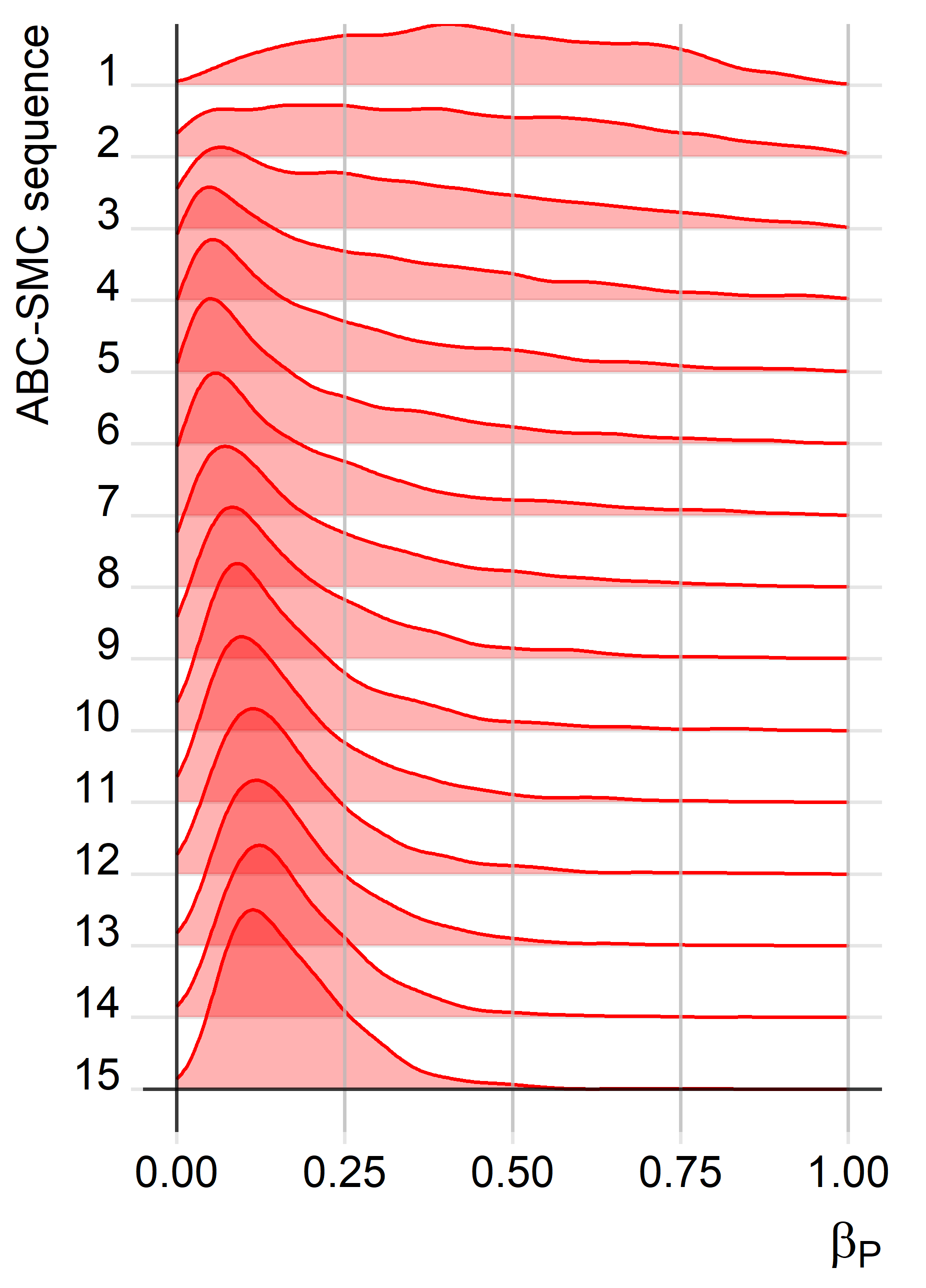


Figure S2. Evolution of $\beta_{P}$ values over 15 ABC-SMC sequences. The mode (95% highest posterior density range) of $\beta_{P}$ in the final sequence was 0.11 (0.03 ~ 0.34).


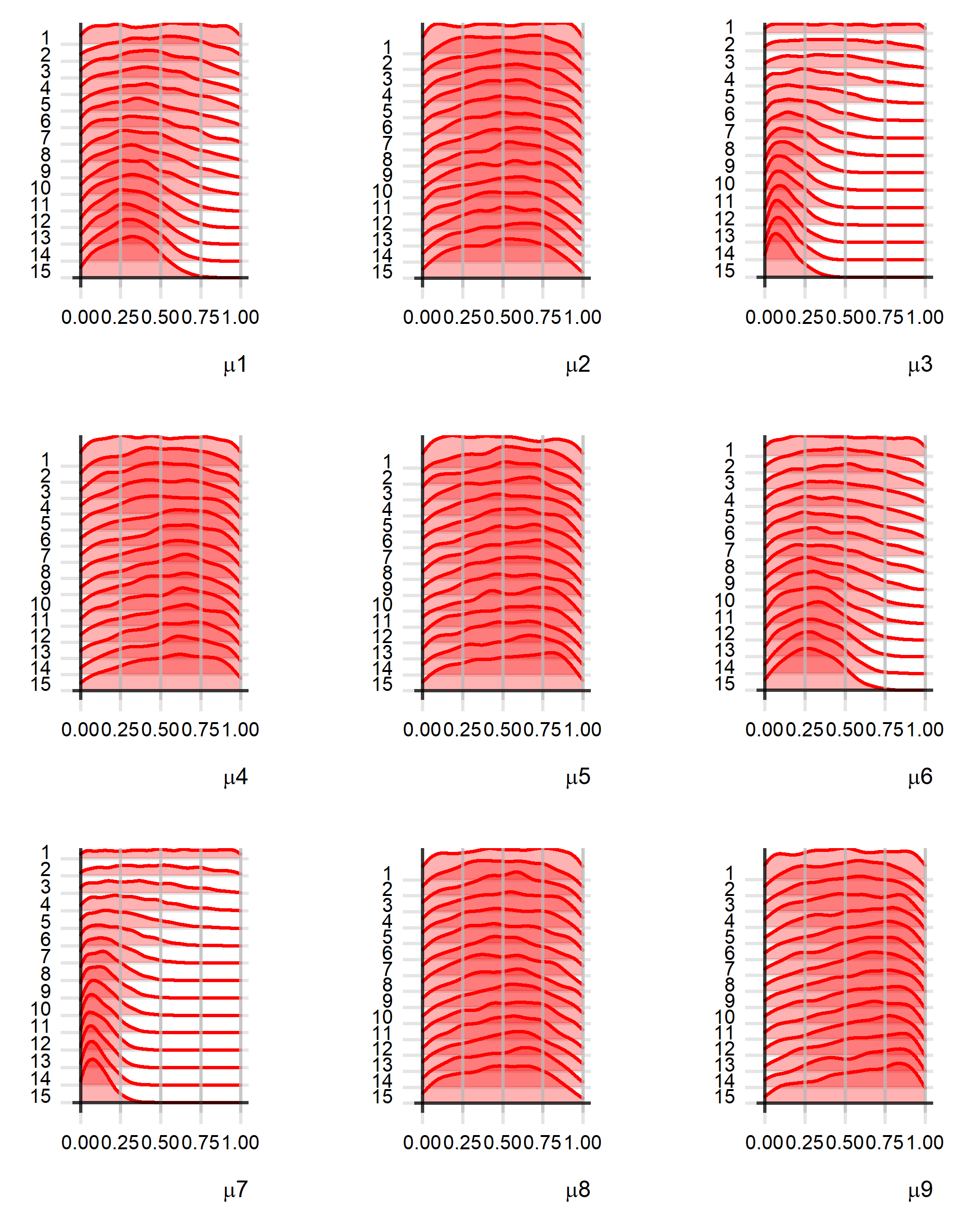


Figure S3. Evolution of $\mu$ values for 9 studied farms over 15 ABC-SMC sequences.


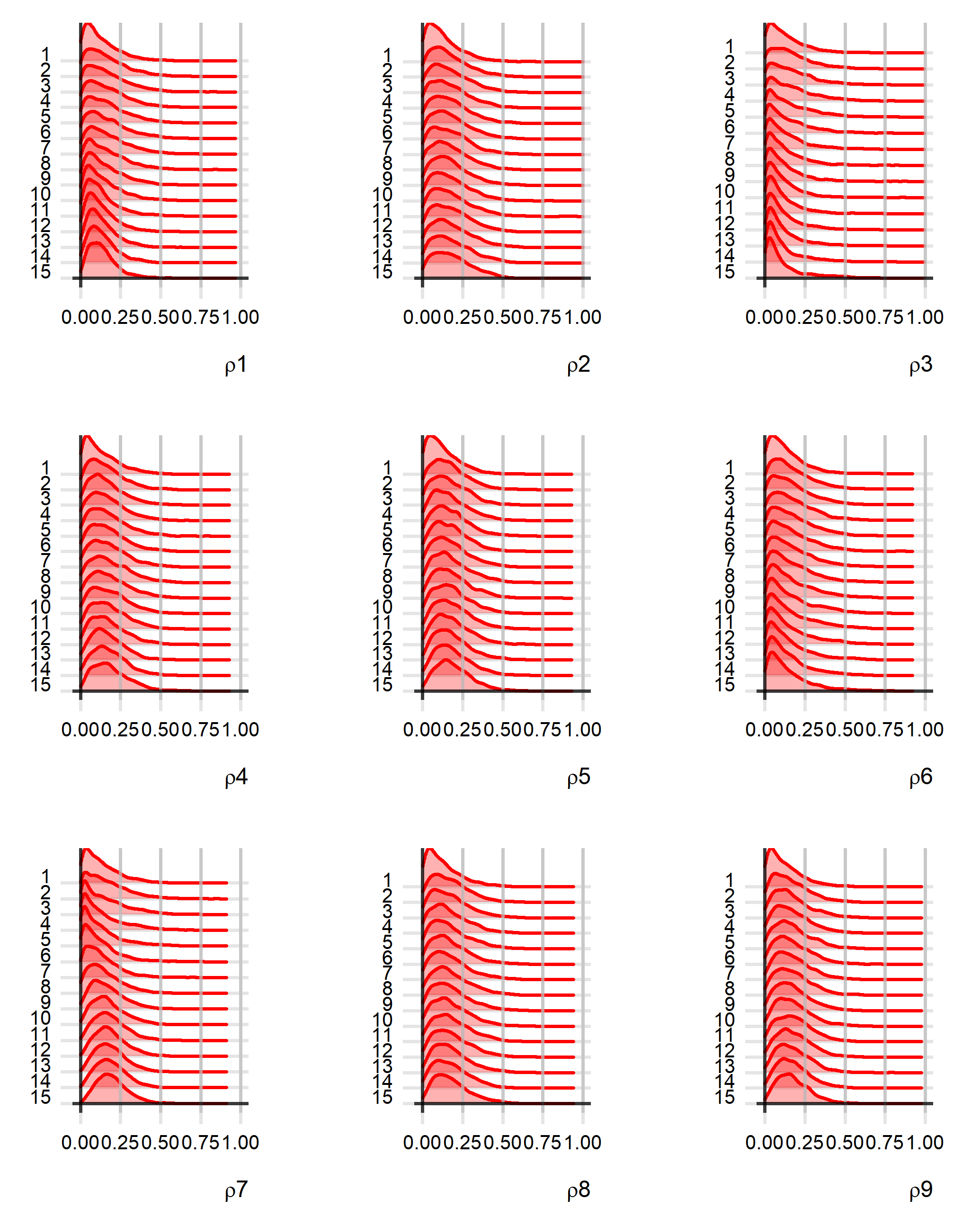


Figure S4. Evolution of $\rho$ values for 9 studied farms over 15 ABC-SMC sequences.


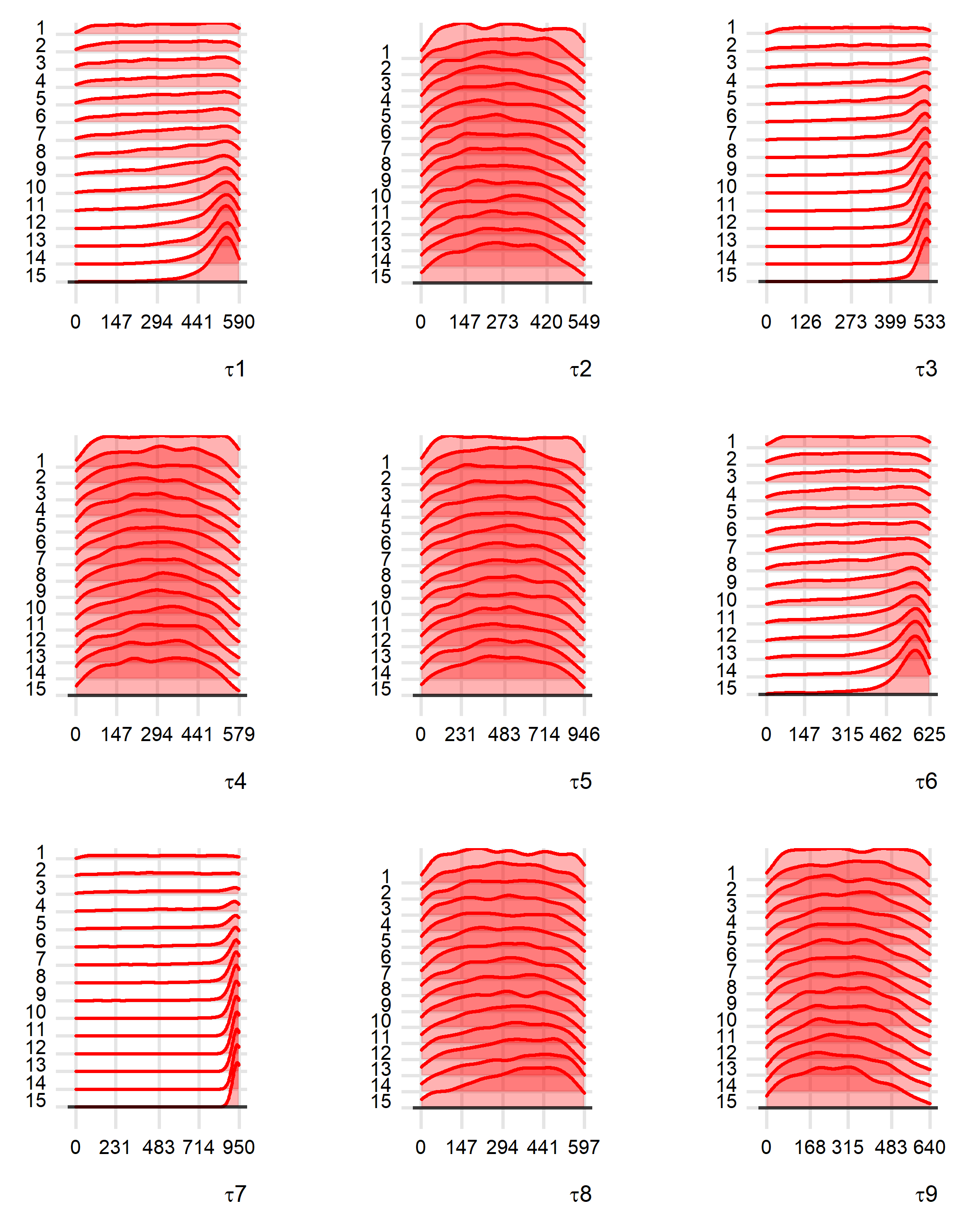


Figure S5. Evolution of $\tau$ values for 9 studied farms over 15 ABC-SMC sequences.

##
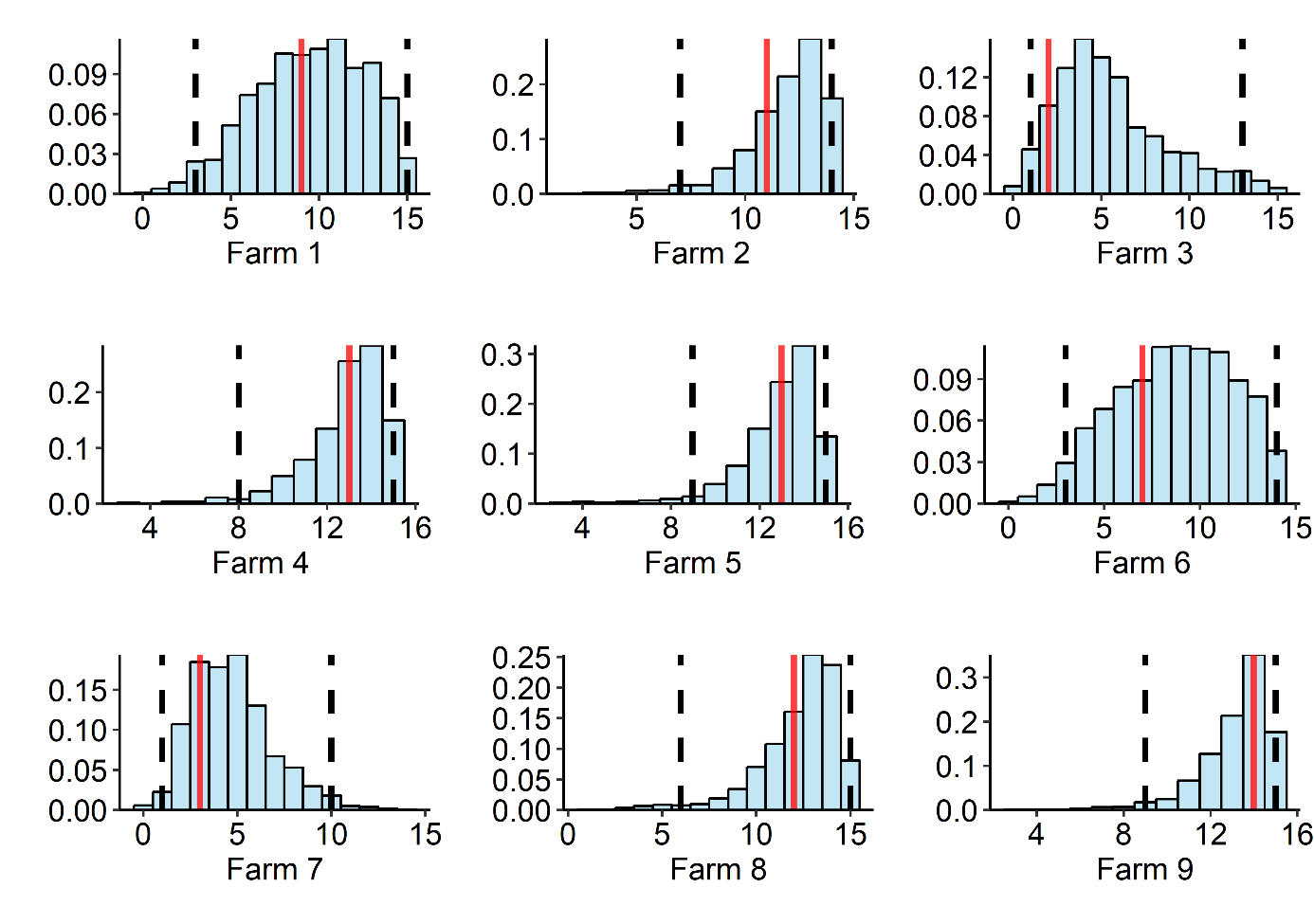


Figure S6. Observed (red line) and simulated (blue histogram) number of test positive heifers in the first sampling round. The distribution of simulated summary statistics was drawn by applying 2000 randomly sampled parameter values from the relevant posterior distributions. Black dashed lines indicate the 95% prediction interval of the simulated distribution.


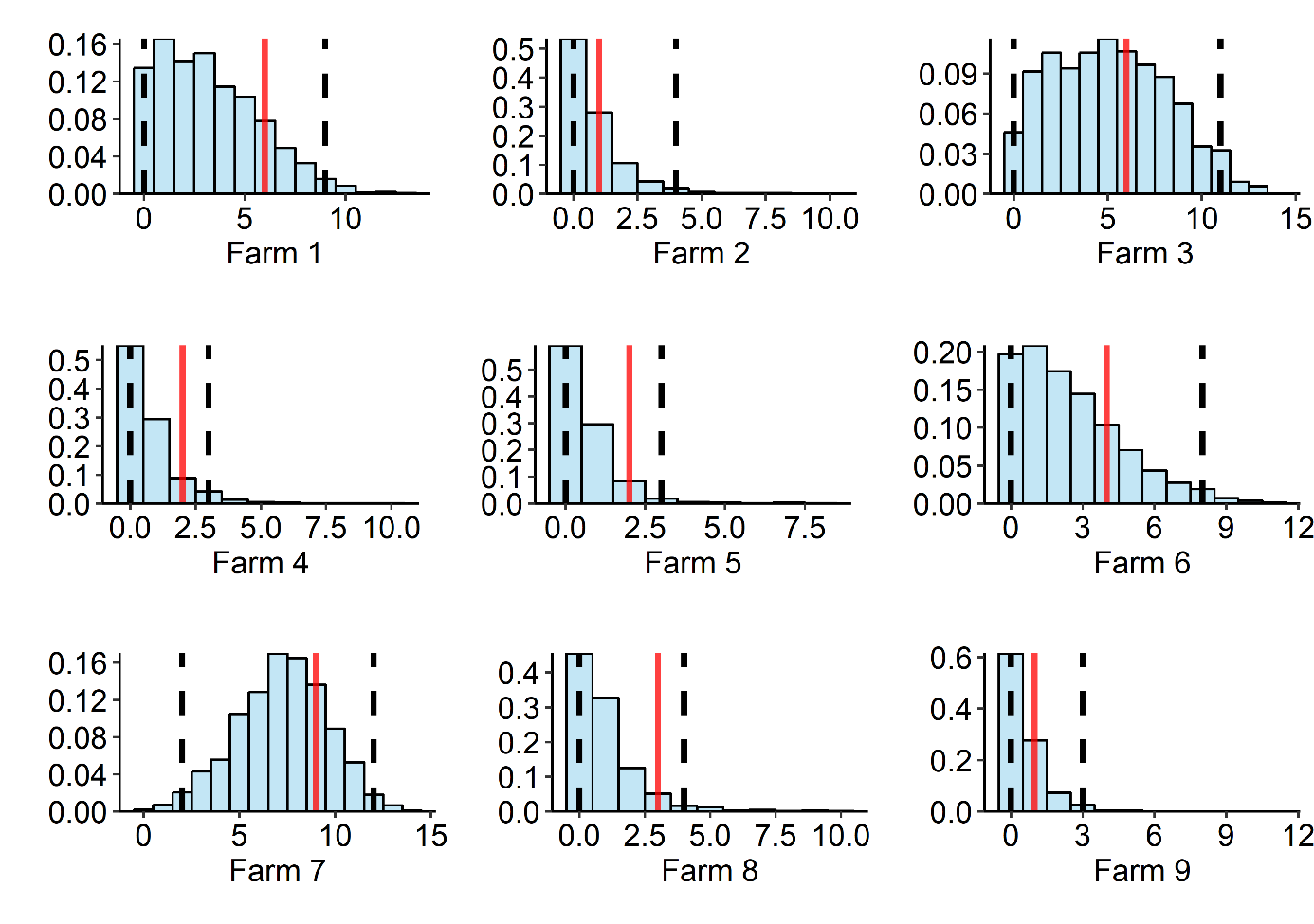


Figure S7. Observed (red line) and simulated (blue histogram) number of seroconverted heifers in the second sampling round. The distribution of simulated summary statistics was drawn by applying 2000 randomly sampled parameter values from the relevant posterior distributions. Black dashed lines indicate the 95% prediction interval of the simulated distribution.
