# Supplementary material for: Estimation of the within-herd transmission rates of bovine viral diarrhoea virus in extensively grazed beef cattle herds
Source: Vet Res. 2019 Nov 29;50:103. doi: 10.1186/s13567-019-0723-2 (PMC6884759; doi:10.1186/s13567-019-0723-2)
Supplement: Supplementary file 7 — Additional file 7. Sensitivity analysis. [file 13567_2019_723_MOESM7_ESM.docx]

## Additional file 7 Sensitivity analysis. We investigate the impact of using a uniform prior of the proportion of introduced PI animals ($\pi\left( \rho\right)$) in this study. Given the purpose was to measure the impact of uniform prior, we collected 1000 particles for each parameter for the sake of computational efficiency.

Mode (95% highest posterior density region) of estimated parameters with different priors of the proportion of introduced PI animals. Rightmost column shows the posterior values of parameters in the original study.

| **Parameters** | $\boldsymbol{\pi(\rho)=}\boldsymbol{U}\mathbf{(0,1)}$ | $\boldsymbol{\pi(\rho)=}\boldsymbol{N}\mathbf{(0.3,}\mathbf{0.1}^{\boldsymbol{2}}\mathbf{)}$ | $\boldsymbol{\pi(\rho)=}\boldsymbol{B}\mathbf{(1.14,7.79)}$ |
| --- | --- | --- | --- |
| $\beta_{P}$ | 0.21 (0.03, 0.75) | 0.09 (0.02, 0.44) | 0.11 (0.03, 0.34) |
| $\mu_{1}$ | 0.42 (0.05, 0.66) | 0.39 (0.03, 0.65) | 0.32 (0.00, 0.58) |
| $\mu_{2}$ | 0.54 (0.12, 0.94) | 0.55 (0.04, 0.88) | 0.53 (0.05, 0.93) |
| $\mu_{3}$ | 0.04 (0.00, 0.24) | 0.06 (0.00, 0.29) | 0.07 (0.00, 0.29) |
| $\mu_{4}$ | 0.72 (0.19, 0.98) | 0.58 (0.13, 0.99) | 0.62 (0.10, 1.00) |
| $\mu_{5}$ | 0.72 (0.15, 1.00) | 0.39 (0.05, 0.96) | 0.80 (0.07, 0.98) |
| $\mu_{6}$ | 0.22 (0.00, 0.53) | 0.14 (0.00, 0.57) | 0.26 (0.00, 0.56) |
| $\mu_{7}$ | 0.03 (0.00, 0.25) | 0.04 (0.00, 0.24) | 0.08 (0.00, 0.25) |
| $\mu_{8}$ | 0.59 (0.11, 0.94) | 0.18 (0.02, 0.89) | 0.48 (0.00, 0.88) |
| $\mu_{9}$ | 0.82 (0.26, 1.00) | 0.87 (0.19, 1.00) | 0.84 (0.09, 0.98) |
| $\rho_{1}$ | 0.14 (0.02, 0.89) | 0.18 (0.04, 0.47) | 0.11 (0.01, 0.32) |
| $\rho_{2}$ | 0.49 (0.02, 0.89) | 0.29 (0.09. 0.55) | 0.10 (0.00, 0.42) |
| $\rho_{3}$ | 0.93 (0.09, 1.00) | 0.36 (0.02, 0.55) | 0.03 (0.00, 0.40) |
| $\rho_{4}$ | 0.25 (0.04, 0.88) | 0.39 (0.06, 0.50) | 0.15 (0.00, 0.39) |
| $\rho_{5}$ | 0.27 (0.01, 0.83) | 0.26 (0.08, 0.53) | 0.14 (0.00, 0.39) |
| $\rho_{6}$ | 0.65 (0.78, 1.00) | 0.26 (0.00, 0.58) | 0.04 (0.00, 0.41) |
| $\rho_{7}$ | 0.24 (0.02, 0.59) | 0.22 (0.12, 0.49) | 0.16 (0.03, 0.37) |
| $\rho_{8}$ | 0.31 (0.02, 0.89) | 0.27 (0.06, 0.51) | 0.12 (0.00, 0.40) |
| $\rho_{9}$ | 0.23 (0.01, 0.86) | 0.28 (0.07, 0.49) | 0.15 (0.01, 0.38) |
| $\tau_{1}$ | 567 (109, 590) | 589 (426, 590) | 546 (359, 590) |
| $\tau_{2}$ | 378 (33, 525) | 167 (5, 495) | 252 (13, 499) |
| $\tau_{3}$ | 525 (161, 533) | 516 (489, 533) | 525 (380, 533) |
| $\tau_{4}$ | 315 (10, 525) | 339 (2, 508) | 210 (24, 530) |
| $\tau_{5}$ | 483 (28, 867) | 799 (27, 872) | 378 (18, 866) |
| $\tau_{6}$ | 609 (45, 622) | 613 (94, 625) | 588 (225, 625) |
| $\tau_{7}$ | 945 (925, 950) | 936 (916, 950) | 945 (913, 950) |
| $\tau_{8}$ | 420 (27, 578) | 152 (57, 587) | 357 (53, 597) |
| $\tau_{9}$ | 378 (13, 567) | 285 (2, 548) | 294 (9, 543) |
| Key: $\pi(\cdot)$, prior distribution; B, beta; U, uniform; N, normal; $\rho$, the proportion of introduced PI animals | | | |

Compared to the original study, some but not all parameters showed different posterior distributions. Especially, the posterior distribution of $\rho$ was highly affected by the prior distribution of itself, indicating that $\rho$ and other parameters were not identifiable under current modelling setting (i.e. data and/or model structure). Along with the preliminary sensitivity analysis (Additional file 4), this result implied that the accurate estimation of parameter values in this study was generally dependent upon the precise information of $\pi\left( \rho\right)$. A recent anecdotal report showed that the prevalence of PI animals within a cattle herd in New Zealand varied between 2% and 12%, and we believe it is highly unlikely to have more than 20% of new-born calves being PI animal under New Zealand farming context. Therefore, we argue that the estimated parameter values based on $\pi\left( \rho\right)$ of our original study were valid estimates.
